# Exploring fetal brain tumor glioblastoma symptom verification with self organizing maps and vulnerability data analysis

**DOI:** 10.1038/s41598-024-59111-6

**Published:** 2024-04-16

**Authors:** Suresh Kumar Natarajan, Jayanthi S, Sandeep Kumar Mathivanan, Hariharan Rajadurai, Benjula Anbu Malar M.B, Mohd Asif Shah

**Affiliations:** 1 School of Computer Science and Engineering, JAIN (Deemed-to-be University), Ramanagara, India; 2 Department of Information Technology, Guru Nanak Institute of Technology, Ibrahimpatnam, Hyderabad, Telangana India; 3 School of Computer Science and Engineering, Galgotias University, Greater Noida, 203201 Uttar Pradesh India; 4 School of Computing Science and Engineering, VIT Bhopal University, Bhopal–Indore Highway Kothrikalan, Sehore, MP India; 5 School of Computer Science Engineering and Information Systems, Vellore Institute of Technology, Vellore, Tamil Nadu India; 6 Kebri Dehar University, Kebri Dehar, 250, Somali Ethiopia; 7 Centre of Research Impact and Outcome, Chitkara University Institute of Engineering and Technology, Chitkara University, Rajpura, 140401 Punjab India; 8 Division of Research and Development, Lovely Professional University, Phagwara, 144001 Punjab India

**Keywords:** Time attribute extraction, Max rationalizing, Fetal brain tumor, Glioblastoma, Min rationalizing, Segmentation, Medical research, Health care, Medical imaging, Public health

## Abstract

Brain tumor glioblastoma is a disease that is caused for a child who has abnormal cells in the brain, which is found using MRI “Magnetic Resonance Imaging” brain image using a powerful magnetic field, radio waves, and a computer to produce detailed images of the body's internal structures it is a standard diagnostic tool for a wide range of medical conditions, from detecting brain and spinal cord injuries to identifying tumors and also in evaluating joint problems. This is treatable, and by enabling the factor for happening, the factor for dissolving the dead tissues. If the brain tumor glioblastoma is untreated, the child will go to death; to avoid this, the child has to treat the brain problem using the scan of MRI images. Using the neural network, brain-related difficulties have to be resolved. It is identified to make the diagnosis of glioblastoma. This research deals with the techniques of max rationalizing and min rationalizing images, and the method of boosted division time attribute extraction has been involved in diagnosing glioblastoma. The process of maximum and min rationalization is used to recognize the Brain tumor glioblastoma in the brain images for treatment efficiency. The image segment is created for image recognition. The method of boosted division time attribute extraction is used in image recognition with the help of MRI for image extraction. The proposed boosted division time attribute extraction method helps to recognize the fetal images and find Brain tumor glioblastoma with feasible accuracy using image rationalization against the brain tumor glioblastoma diagnosis. In addition, 45% of adults are affected by the tumor, 40% of children and 5% are in death situations. To reduce this ratio, in this study, the Brain tumor glioblastoma is identified and segmented to recognize the fetal images and find the Brain tumor glioblastoma diagnosis. Then the tumor grades were analyzed using the efficient method for the imaging MRI with the diagnosis result of partially high. The accuracy of the proposed TAE-PIS system is 98.12% which is higher when compared to other methods like Genetic algorithm, Convolution neural network, fuzzy-based minimum and maximum neural network and kernel-based support vector machine respectively. Experimental results show that the proposed method archives rate of 98.12% accuracy with low response time and compared with the Genetic algorithm (GA), Convolutional Neural Network (CNN), fuzzy-based minimum and maximum neural network (Fuzzy min–max NN), and kernel-based support vector machine. Specifically, the proposed method achieves a substantial improvement of 80.82%, 82.13%, 85.61%, and 87.03% compared to GA, CNN, Fuzzy min–max NN, and kernel-based support vector machine, respectively.

## Introduction

Brain tumor glioblastoma is the formation of brain cells, and it can begin elsewhere while spreading in the brain. The process of functioning of the tissue in the surrounding area of the brain has been detected. It is important to detect and identify the Glioblastoma in children as it presents distinct challenges compared to adult cases, as the children have developing brains and bodies, making treatment decisions more complex. Understanding the specific characteristics and factors associated with pediatric glioblastoma is essential for providing tailored and effective treatment strategies. Thus, analyses of pediatric glioblastoma provide valuable insights into treatment optimization. It helps in determining the most suitable therapeutic approaches, including surgery, radiation therapy, chemotherapy, immunotherapy, and targeted therapies. Monitoring the tumor's response to treatment allows healthcare professionals to make informed decisions about adjusting or switching treatment modalities to achieve the best possible outcome. The methods of indicating the function of the brain tissues have been analyzed to find the brain tumor glioblastoma. Many men and women have been affected by brain tumor glioblastoma, which is caused by gender, age, family history, ionizing radiation, etc. the first sign of brain tumor glioblastoma are seizures and speech and vision problems. This tingling and numbness manage the hands, legs, feet, face, and arms. This can be checked using MRI images, which examine the checking of relaxes’ hearing, vision, and coordination. Also, a neurological examination is done for the abnormal tissues. In this approach, managing the rapid and serious health decline addresses the causes of the severe problem without problems. Some men and women experienced sharp pain in the specific area to analyze the sneezing and coughing. Some brain-related issues are identified by using MRI images. The brain and spinal cord make the worst time, which happens suddenly in all Childs. The process of managing the brain tumor glioblastoma is carried out using medication and meditation. This can be identified by the method of max weber’s rationalization for replacing the motivators and the behavior of the society and the reason for the minimum effect for the concept of a specific reason. These are the process of managing stress, and this is the cause in the form of rationalizations.

The mood of certain Childs and the morning commute for managing the disappointment and the responsibility of the defective theory. The depression-based anxiety and the sudden factor for analyzing the development of cancer-based headache is the form of brain tumor glioblastoma. The boosted decision time attribute extraction manages the stress level in people’s behavior. This reason for causing the appear good factor for the creditable element and the cruel behavior of the process, act, and the rationalizing process is being demanded using the most attraction of the system is proposed. Some of the rationalizations are predictability, calculability, and dehumanization. This is invented by the factor for affecting the lightness of the people's mindful stress. The term attribute extraction is done using machine learning to extract the free-form attributes for describing the product attributes. This time attribute manages the child’s attribute. It enables the factor for spatial attribute and the extraction time to produce the attribute of the mentally disabled. In this juncture, the classification and the image segmentation have been used for finding the brain tumor glioblastoma and the formation of the tissues in the brain images. The specific research problem can be defined as the need to improve the accuracy and efficiency of glioblastoma diagnosis using MRI brain images and to develop effective techniques for segmenting and recognizing the tumor in order to facilitate timely and accurate treatment. These images can be analyzed using MRI images, and the brain tumor glioblastoma factor for the recognition of the images is done. Since in our opinion it is proposed technically and not clinically tested, we accept your opinion and humbly request you to allow us to withdraw the word guaranteed detection of glioblastoma from our research paper and replace it with the word Diagnosis of glioblastoma. Through this article, we inform you that these changes will not change depending on the technology and their policies and we take responsibility for any future questions that may arise due to this. As we said, we have deleted the word guaranteed detection of glioblastoma and replaced it with the word diagnosis of glioblastoma. Please note that this line has been replaced at position 35 in our research paper. This is found primarily in people who are in the stress field. The accuracy and efficiency of the data are done using the rationalization of the system. The contribution of the paper is as follows:

The fundamentals of diagnosis from brain tumor glioblastoma diagnosis were analyzed.

- Medical field finds high-quality fetal brain tumor caustic diagnostic equipment as it is adapted to some future technological variations and technological developments.
- The research proposes a novel approach by combining self-organizing maps, which are unsupervised learning algorithms used for clustering and visualization, with vulnerability data scanning techniques. This integration allows for the detection and verification of fetal brain tumor glioblastoma symptoms in an innovative and potentially more effective manner.
- To access the risk assessment process utilizing the vulnerability routine data scanning to decrease the vulnerability by scanning and threat assessment.
- This research aims to improve diagnostic accuracy, enable early detection and intervention, and enhance the understanding of fetal brain tumor glioblastoma symptoms through the use of self-organizing maps and vulnerability data scanning techniques.

The significant contribution and novelty of the paper is summarized below,

- A proposal is formulated to enhance the performance of medical diagnostic equipment in detecting fetal brain tumor symptoms. This proposal ensures adaptability to future technological advancements in the field.
- Introducing a novel approach, the paper combines self-organizing maps with vulnerability data scanning techniques. This integration offers a fresh perspective on detecting and verifying fetal brain tumor glioblastoma symptoms, potentially leading to more effective diagnostic outcomes.
- Access control management strategies are proposed to regulate security levels in the risk assessment process, leveraging vulnerability data scanning to mitigate potential risks. This innovative approach has resulted in the development of a groundbreaking method for nonlinear dynamics analysis using visibility graph techniques.
- The primary objective of this research is to enhance diagnostic accuracy, facilitate early detection and intervention, and advance the understanding of fetal brain tumor glioblastoma symptoms through the innovative integration of self-organizing maps and vulnerability data scanning techniques.

All research focus mentioned above indicate how glioblastoma diagnosis is made. Through this^1–3^, the existing research paper Curcumin-based-fluorescent probes targeting ALDH1A3 as a promising tool for glioblastoma precision surgery and early diagnosis explains in detail how it works within a framework. Based on this explanation, in our research paper we have extended our research within the framework of this glioblastoma diagnosis machine learning methods.

The sectional discussion manages as follows: Section “Introduction” describes the overall process of the paper in the introduction format, and in Section “Literature review”, the existing document has been reviewed as a Literature review. In Section “Boosted division time attribute extraction (BDTAE)”, the methods have been elaborated by involving the proposed system. In Section “Results and discussion”, the results have been executed; in Section “Conclusion”, the conclusion regarding the paper has been written with future work.

## Literature review

Das et al. study discussed the brain tumor segmentation that has been analyzed using artificial intelligence based on the neural network. In this instance, the process of managing the brain tumor segmentation strategy using PRISMA. Also, the quantitative and qualitative analysis compares based on the AI models. So, the ranking method for the estimations has been adopted for the computation uses^4^. The risk of images MRI is used in AI-based clustering. Also by implementing this in the currently proposed system the brain tumor is found using this segmentation method. The risk of bias for lowering BTS is proposed.

Nehra et al. maintain the Nano biotechnology manner for the therapies for efficient nano-system which manage BBB brain cancer by monitoring the brain tumor. In their article, the growth of the Nanomedicine and the electro-magneto optical nano-system has been proposed using the smart electro-magneto optical nano-system for tumor optimization. Photodynamic therapy and advanced brain cancer treatment manage the nano-system from the brain tumor classifications. The high-efficiency resistance of brain cancer has been prescribed for the thermostat. Also, by involving his methods in the currently proposed system, the brain tumor can be personalized by the cell therapy treatment^5^.

Mormann et al. study discussed the rationalization and the organizational factor for responding to the societal for managing the employment for the marketization of the processing values is proposed. The process of managing the rationalization of the organizational principle factor has been produced for the individual contents for the meaningful value in the organization is presented. Also, the comparative study which manages the individualization and the marketization will follow among the organization. Involving this in the currently proposed system, the brain function and the system’s formation to track the individual factor have been produced^6^.

The neurological disease, which increases in the number of different factors to produce the polymerization of the behavior of the patients affected by the brain diseases, is proposed^7^. This method of rationalization, the MSG and the aggregation of the human psychological factor have been analyzed using brain images. The process of segmenting and the classification of the behavior of the people is explored. Then the brain tumor was diagnosed. The process of managing the protein aggregation and the neurological factor accepts the people’s behavior and controls the neurological behavior. By involving this in the currently proposed system, the neurological factor for the secondary structure has been submitted by Ahanger et al. (2021).

Lu et al. study discussed managing the immune and genes for infiltration and recognizing neuro-related problems. The characterization of the IRG classifies the hydrocortisone and the other diseases which contribute to the efficient immunology status. The surviving factor for improving the patient outcomes and the individual health analysis is done. The process of classifying the IRG-related factor for the poor fetal rate and the disease diagnosis is proposed. The high and low risk of the IRG has been done using several cytokines and the validation of the results of the specific therapy. Also, by implementing this in the currently proposed system, the personalized factor for diagnosing the model of the IRG classifier is presented^8^.

Villanueva et al. study discussed the critical decision to manage the daily business for performing the attributes’ accuracy and integrity. The classification of the nonparametric factor has been proposed using the abnormality of the segmenting of the images of the diseases, and the decision-making has been presented. The process of managing the accuracy and the execution time has happened for the detection of the event, and the clustering technique has been done. This parametric sequence of the data and the results found with the results and the faulty sensors has been done. Also, by involving this in the currently proposed system, the decision-making according to the MRI images has been done to produce the classification of the images^9^.

Zhao et al. research discussed the ultrasound of the Dolichos bean protein for UAE increased the improvement of the functions and the formation of the system to produce the bean protein’s antioxidant factor, which increases the dolichols protein. The DLP structure has been proposed using the function of the different types of diseases. The process of managing the industrial factor and the extraction of stability has been submitted. Involving this method in the currently proposed system, the functional properties which manage the suitability for the primary and the second factor, which consists of the ultrasound extraction, have been presented^10^.

From the above consideration, this research paper gets a clear answer when practical testing is conducted with equipment in the medical field depending on human organs. By analyzing the research papers which are similar to the proposed system to detect and identify the brain tumor glioblastoma disease by image extraction process by segmentation of image with different methods to find an effective method. From that answer we can clearly explain how the standardization of positioning is captured in the analysis of the embryo for this mechanical processing method that we propose. Because the images obtained here always represent different stages of the embryo due to the different step methods of the samples taken. In the ten types of images we have taken, when using all its data processes, they take up more pages, so we have described 10 processes for each stage, 10 processes for each stage, through different images. It is because of this that our conceptual research is presented in different static fragments of captured images.

The following are some significant summary limitations of existing methods: many existing methods suffer from limited accuracy, which can lead to suboptimal results and hinder their practical applicability in real-world settings. Some methods exhibit high computational complexity, making them impractical for applications requiring real-time processing or large-scale data analysis. This can pose challenges in resource-constrained environments or systems with strict performance requirements. Certain methods are tailored to specific data modalities, such as MRI or CT scans. While effective for the targeted modality, these methods may struggle to generalize across different imaging modalities or lack robustness when applied to diverse datasets. Noise and artifacts commonly present in medical images can adversely affect the performance of existing methods. Many approaches are sensitive to noise and may produce inaccurate results or require additional preprocessing steps to mitigate these effects. Despite achieving promising results in controlled experiments or specific datasets, some methods may struggle to generalize to unseen data or diverse patient populations. This limits their utility in clinical practice and highlights the need for more robust and generalizable solutions. Interpretability is crucial in many applications, where decisions directly impact patient care or diagnostic outcomes. However, some methods lack transparency and interpretability, making it challenging to understand their decision-making process or trust their results.

## Boosted division time attribute extraction (BDTAE)

BDTAE is a method used for extracting diagnosis using time attributes. The images are split based on the time using the octagon-based recognition method. The raw collected data are directly taken for feature extraction. Then, the image’s dimensionality is reduced after applying the recognition method with time and division line concerning the spot present inside the region of the shape extracted. This method is used to reduce the error occurring by manual analysis for finding the presence of a tumor when the size of the cancer is small and cannot be viewed by humans or the company of frequency shades that might look similar to cancer by guaranteeing to spot the location so, automatic recognition of images based on an octagon-based structure is adopted^11^.

The choice of the octagon-based recognition method in our research is grounded in several key considerations, each contributing to the effectiveness and suitability of this approach for the specific task of image classification and recognition. The octagon finds a balance between intricacy and flexibility, allowing for effective identification in a variety of real-world scenarios. It is superior over simpler shapes such as squares or circles because of its tolerance to changes in size, rotation, and partial occlusion. Because of the arrangement of its eight vertices and edges, it has a distinct visual style that makes it easy to categorize even in settings with minor visual differences. This reduces its sensitivity to noise and tiny aberrations in the input images. This capability is especially useful in noise-prone applications like as surveillance systems and medical imaging. Furthermore, octagon-based recognition algorithms are more computationally efficient than more sophisticated shape descriptors, making them appropriate for real-time or resource-constrained applications that need quick processing of huge amounts of visual data. Previous research has shown that octagon-based recognition approaches are successful for a variety of image analysis tasks, including as pattern identification, object detection, and scene understanding. By embracing our combined thoughts and achievements in the field, we may increase the performance and dependability of our proposed approach while leveraging this well-established foundation.

**Figure 1:**
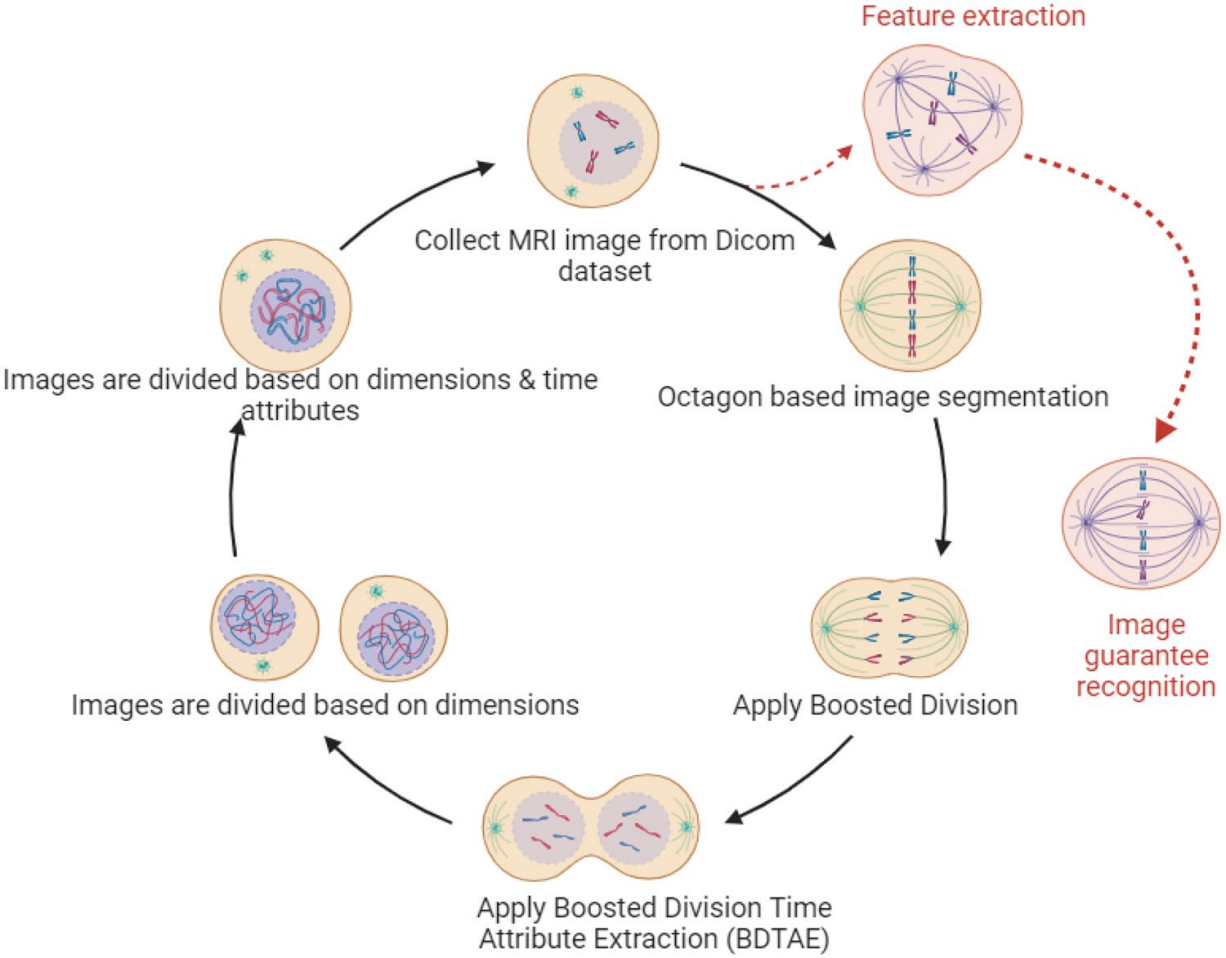
BDTAE-based diagnosis feature extraction.

Analyzing the entire image for a spot took a long time to find a diagnosis place in a vision based on a time attribute adopted on the octagon structure for extracting the feature of the tumor from the input. Thus, the shape or design of the octagon should be higher than the image to fix the image inside the structure for dividing the image based on different conditions and times^12^. This process reduces the time complexity for feature extraction and points out the tumor’s exact location. The flow of BDTAE-based Diagnosis feature extraction is shown in Fig. 1. To identify the exact spot of the brain tumor glioblastoma, octagon-based segmentation of the image is adopted. That is, the sample brain image is placed inside the octagon structure and made the image into a division based on the attribute time and dimensions. The spot detected part after division is extracted as a feature of the tumor by image processing; thus, the output of this method is a brain tumor glioblastoma along with noise data by image guarantee recognition which is when all the entities and attributes state the extracted data is entirely responsible for image recognition^13,14^.

The BDTAE (Boosted Division Time Attribute Extraction) is a method used for extracting Diagnosis using time attributes. Initially, each input image is treated for recognition and isolating for detecting the brain tumor spot from the samples containing the very micro-location on the entire sensed image. The input image is conceived as the octagon diagonal region. Humans can’t view the micro-location of the tumor, so we moved to automatic recognition of image-based on an octagon-based structure. Hence, the glory and isolation for detecting the brain tumor spot using the octagon diagonals sectionalization method. The guarantee spotting processes identify and separate the unmatched brain tumor spot and the matched brain tumor spot. The brain image is segmented, and diagnosis recognition is used to check segmented brain images. When the recognized location is accurate, we can realize the exact point of the brain tumor. BDTAE always has specified procedural aspects to process the brain image with features such as boundary, resolution, space, and density. These components are identically complex to adulterate from brain image navigation.

**Figure:**
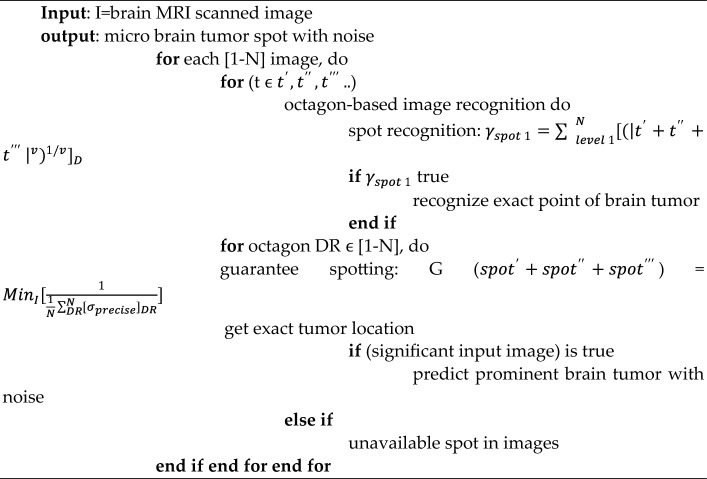
Algorithm: The algorithm for boosted division time attribute extraction.

To overcome these difficulties, the proposed system uses the Boosted Division Time Attribute Extraction (BDTAE) image recognition guarantee based on a time attribute adopted on the octagon structure for extracting the feature of the tumor from the input. The guarantee spotting detects the exact location of the significance in the input image. Then we get the prominent brain tumor spot^15^. The output of this method is a brain tumor along with noise data by image guarantee recognition which is when all the entities and attributes state the extracted data is entirely responsible for image recognition^16^.

**Figure:**
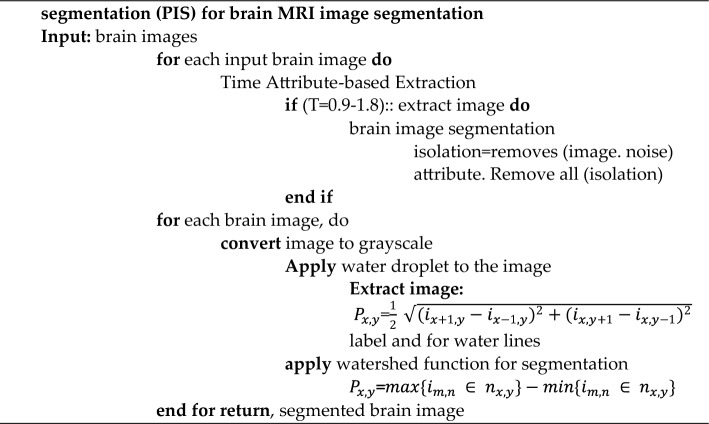
Algorithm: Algorithm for hybrid time attribute extraction (TAE) and partial image.

The Time Attributes Extraction method is used to segment the brain image to remove the noise based on the time attributes. The brain image is filtered during the segmentation process after feature extraction based on TAE and Partial Image Segmentation (PIS); thus, the segmentation is known as the Hybrid TAE–PIS method. This hybrid method segments the image to find the distinct object, and the partial segmentation method removes the image’s noise. The watershed algorithm is used for segmenting the image; before that, the idea is converted into a grayscale image similar to the catchment basin of a brain tumor region. The water droplets are enforced on a brain image’s ramp for segmenting a specified area of an idea for Diagnosis of a tumor in an MRI brain image^17,18^.

### Noise reduction from an image using segmentation

**Figure 2:**
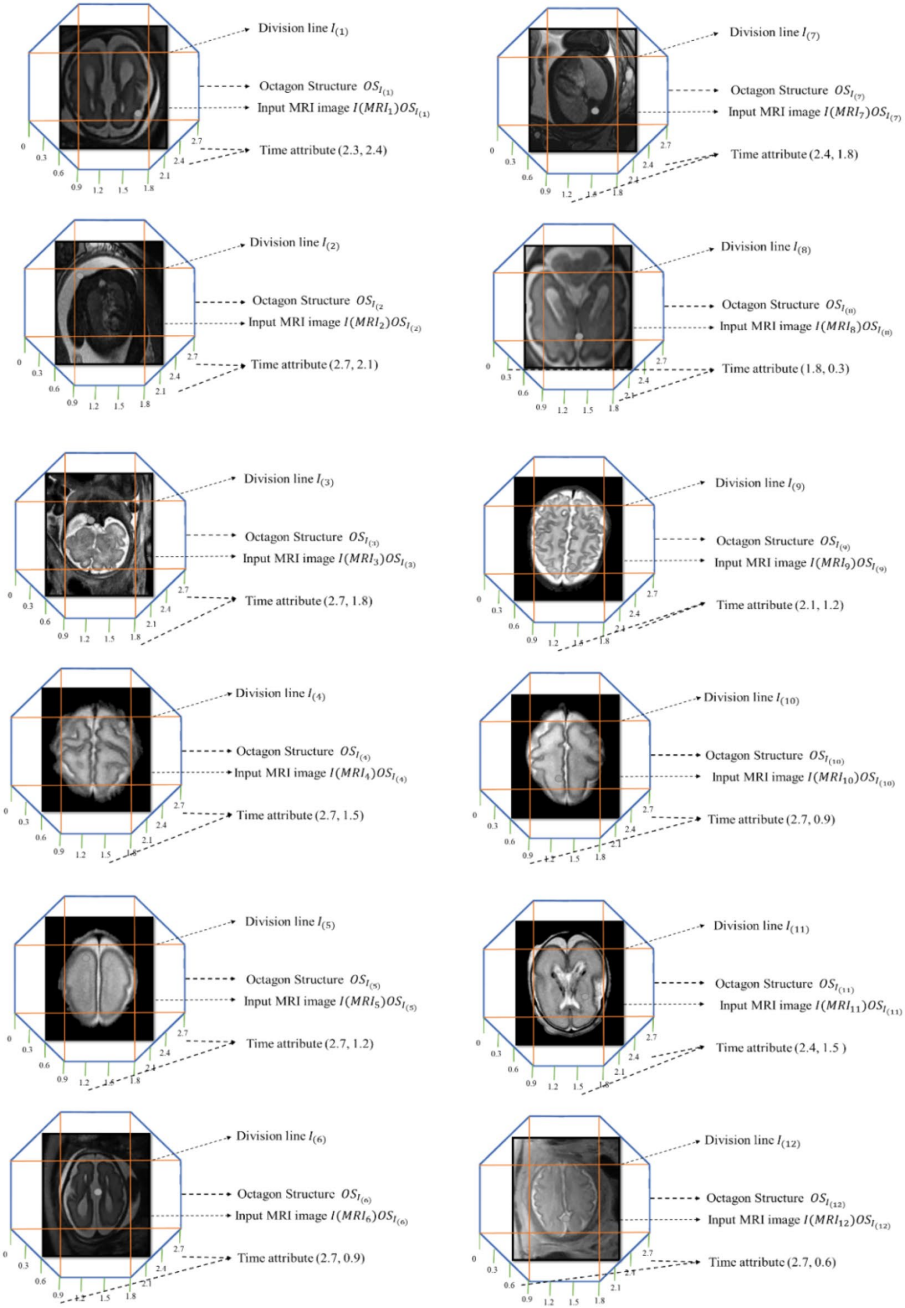
Time attribute-based extraction.

Image segmentation is a method that segments the digital image into smaller frames to reduce the complexity of the image to extract exact changes found in the image through the image analysis process. In this system, by dividing the image for processing, the unwanted noise is reduced by Time Attributes Extraction (TAE), as shown in Fig. 2.

Normally after data acquisition, the pre-processing of an image is considered for extracting a quality image by reducing frequencies or noise from pixels of an image^19^. Still, in the proposed system, raw data before any preprocessing is taken as input to the system because there might be a chance of removing a small tumor spot present in the image due to filtering, so the noise removal process is done after the feature extraction process by TAE. The image in this method is filtered during the segmentation process after feature extraction based on TAE and Partial Image Segmentation (PIS); thus, the segmentation is known as the Hybrid TAE-PIS method^20^.

**Figure 3:**
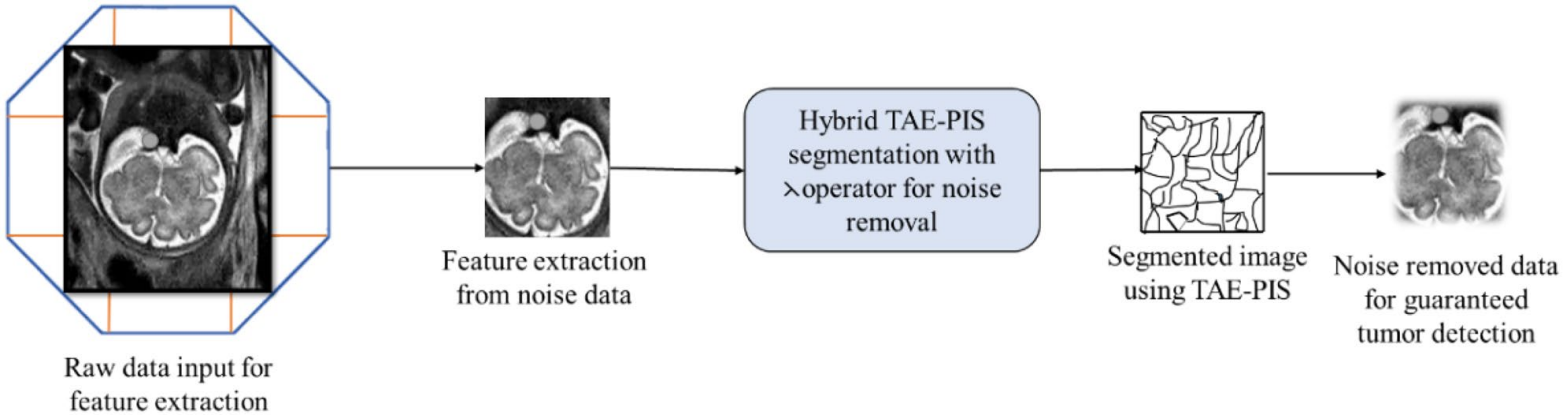
Noise removal by segmentation.

The segmentation of the image into a homogenous region is based on the time attribute for the partial image segmentation process, as shown in Fig. 3. The goal of this segmentation is to find the distinct objects from the image, but the main objective of the partial segmentation method is to remove noise from the image data during the process of segmentation. Thus, this method should be applied to the noisy image and also prevent an image’s edges. There are many methods for segmenting the images partially for noise removal. Still, the widely used method is based on the watershed algorithm, a segmentation method based on the topological interpretation of areas and regions. The grayscale image in a watershed is similar to the catchment basin of a brain tumor glioblastoma region^21,22^. The water droplets were applied on the ramp of an image for segmenting a particular area from an image for diagnosis of a tumor from an MRI image. The region around the tumor region is known as an area of the spot; thus, each point in the region of a tumor point is connected to the extremal end by a monotonic path. The partial segmentation is applied to the feature extracted image is shown as,

1 $${P}_{x,y}= \frac{1}{2} \sqrt{{({i}_{x+1,y}-{i}_{x-1,y})}^{2}+{({i}_{x,y+1}-{i}_{x,y-1})}^{2}}$$

where, i_x,y_—represents image value at range (x,y). The partial segmentation is applied to the regions of the gradient image as the segmented boundaries correspond to the tumor’s edges. A morphological gradient is used for effective segmentation as follows,

2 $${P}_{x,y}=max\left\{{i}_{m,n} \in {n}_{x,y}\right\}-min\left\{{i}_{m,n} \in {n}_{x,y}\right\}$$

where n_x,y_ states the center of the neighborhood on the pixel with x and y regions. In this system, the time division-based image from the octagon structure is the extracted feature based on the dimensions of the image x and y. This partial segmentation generates too many small regions of an image; thus, to remove noise or smoothen the image, an additional ⋋ operator is applied to the segmented image to eliminate noise from the image. Therefore, the operator ⋋ has the process for regional extreme from the image through generating a succession of a smoothed image from feature extracted image repeatedly based on time^23^. Thus, the segmentation process applied to the image with a gaussian noise gets removed. As the operator's effectiveness for smoothing the image over-segmentation is apparent, increasing the ⋋ number of segments by the partial image segmentation method can be controlled. The technique used for segmentation for removing noise helps in extracting quality segmentation of images for extracting diagnosis of a brain tumor glioblastoma without any unwanted noise extracted for recognizing brain tumor glioblastoma^24^.

### Octagon-based image recognition

The image recognition with the structure octagon for establishing diagnosis is by splitting the image with time attributes. In geometry, an octagon is a shape that contains the total number of vertices and edges 8 on a 2-D surface. It is also built with the conditions in geometry like squares, rectangles, and triangles, so this shape is used for image recognition-based tumor identification. Similarly, other geometric shapes have more or fewer sides and vertices than the octagon. In contrast, with fewer sides, it is challenging to recognize the image because of its shape and size; with more sides, the process becomes complex. Thus, octagon-based image recognition is adopted to make the award effective and reduce the complexity of the process^25,26^. Therefore, the size of the octagon should always be larger than the MRI image, for that is S_0_ < I. After fixing the image inside the structure, the edges or sides are connected to form a division line to guarantee the spotting of a brain tumor glioblastoma.

**Figure 4:**
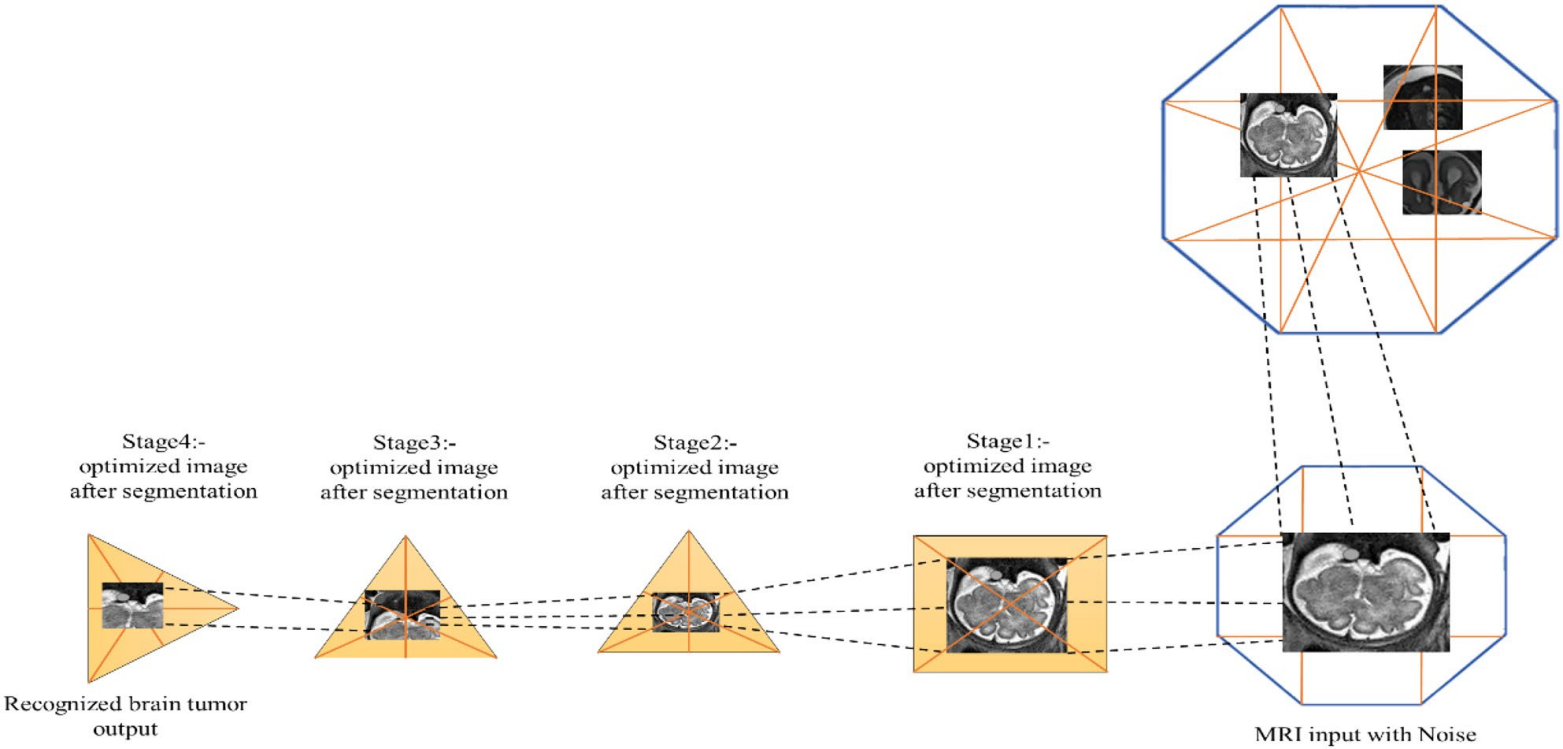
Tumor image recognition using octagon structure.

The image recognition using the octagon structure is shown in Fig. 4. The Dicom file format input image is fixed inside the octagon for extracting the feature of the tumor by dividing the image based on dimensions using the time attribute for stating the recognized image is a diagnosis of brain tumor glioblastoma. The MRI image might contain a very small or micro-level tumor. Considering this state, a division of the image based on time division with the geometric shape is used to recognize the image. The image is not pre-processed before applying the structure to the image. During each image's dimensional segmentation, the noise gets reduced only on the particular segmented portion. The selection of diagonals for segmentation was considered using the octagon structure with the image recognition procedure^27^. The entire image is considered as N, then let the tumor's diameter is 3 mm by extracting cancer from the image, the fewer information diagonals get segmented from an image that is N − 3 mm = n, in this expression, n states the remaining regions without tumor from the entire image N. After segmentation, an optimized segmented diagonal is extracted based on the finest vertex of the image based on the image recognition with the diagonals of an octagon as follows,

3 $${\vartheta }_{Spot}={\Sigma }_{i=1}^{k}{({(\left|{t}{\prime}+{t}^{{\prime}{\prime}}+t{\prime}{\prime}{\prime}\right|)}^{1/u})}_{D}$$

Thus, the diagnosis based on the features of the tumor in the MRI image of stage 1 segmentation is determined using the time attribute. The input image is in the diagonals of the octagon by removing the diagonals of the exact spot of the tumor from vertex s.

4 $$GS\left({s}{\prime}, {s}^{{\prime}{\prime}},{s}^{{\prime}{\prime}{\prime}}\right)= {min}_{I}\left(\frac{1}{\frac{1}{k} {\sum }_{da=1}^{k}{({\gamma }_{p})}_{da}}\right)$$

where regions of the octagon are stated as da, da = 1 to 8 is represented based on the vertices of Diagnosis s. Thus, the spot in the image does not change the precise location of the brain tumor glioblastoma. Still, only the undetected area of the tumor was removed from processing to find the presence of the tumor in the brain image. Similarly, the process continues until a diagnosis in a brain tumor glioblastoma gets recognized from an appearance at different stages. In the first stage, the recognition of the whole image is rationalized based on the dimensions of an idea where some part of the N image gets reduced to n. Now, for the second recognition stage, reduced n is taken as input, and it is rationalized similarly. This process continues until all the un-spotted regions of an image get removed from the N image then the diagnosis brain tumor glioblastoma gets recognized from an MRI image^28^.

The octagon-based Diagnosis method detects the brain tumor spot in the MRI brain image. Time attribute-based segmented brain images are fed into the input of this system. The octagon shape contains the vertices and edges as 8 in two-dimensional surface, which includes the forms of square, triangle, and rectangles used for extracting the brain image for detecting the brain tumor. Initially, I denoted it as a brain image, representing the octagon's size. The brain image is correctly fixed inside the octagon shape when the size of the octogen is greater than the size of the brain image. After the image arrangement inside the octagon, the division line is formed in the brain image by connecting the edges or sides of an octagon^29^.

**Figure:**
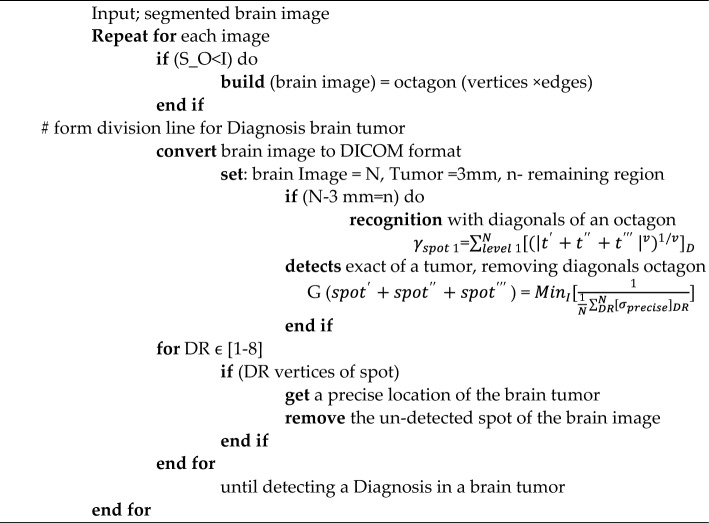
Algorithm: Pseudocode for the process of exact brain tumor detection.

The brain image is converted into DICOM format that is used to fix inside the octagon shape for extracting the feature of the tumor by dividing the image based on dimensions using the time attribute for stating the recognized image is a diagnosis of brain tumor. The brain image is conceived as N, assuming the tumor diameter is 3 mm. For extracting cancer from the brain image as N − 3 mm = n, in this expression n depicts the region without tumor^30^. Based on the finest vertex, the idea is removed and recognized with the diagonals of an octagon as follows,

5 $${\gamma }_{spot 1}={\sum }_{level 1}^{N}{[{({\left|{t}{\prime}+{t}^{{\prime}{\prime}}+{t}^{{\prime}{\prime}{\prime}}\right|}^{v})}^{1/v}]}_{D}$$

The diagnosis based on the tumor in the MRI image is influenced using the time attribute. The input image is in the diagonals of the octagon by removing the diagonals of the exact spot of the tumor from vertex s.

6 $${G(spot}{\prime}+{spot}^{{\prime}{\prime}}+{spot}^{{\prime}{\prime}{\prime}})={Min}_{I}[\frac{1}{\frac{1}{N}{\sum }_{DR}^{N}{[{\sigma }_{precise}]}_{DR}}]$$

When the octagon regions (DR) are stated as DR = 1 to 8 based on the vertices of diagnosis, then the detected spot does not change the precise location of the brain tumor but only removes the un-matched spot of the tumor to find the presence of the tumor in the brain image. Similarly, this process continues until all the un-spotted regions of an image get removed from the brain image. Then the diagnosis of a brain tumor gets recognized from an MRI image^31^. The problem of standardizing the position of planes is important in machine image processing, but we know that you cannot explain how software can process images acquired in different planes. But based on the MRI samples we took and used each model for a separate training purpose. In this way we took five images. While processing those five images, we did one image training while keeping the other four images for testing purposes. As soon as they finish one image, the next image automatically decides their training needs. Today we have discussed how to process images obtained in different planes in this way.

### Max rationalizing and min rationalizing of image

Rationalization is reducing the diagonals of an image from a larger dimension to lower dimensions to recognize a diagnosis tumor from an input image. Thus, maximum and minimum rationalization of ideas helps generate a different state of equilibrium for extracting an output as diagnosis tumor detection.

Minimum rationalization is the total number of inputs working with the median number of images processed for the exact detection of a tumor spot concerning unit time. Then the minimum rationalization is determined using the,

7 $${min}_{R}=Total number of image \times \frac{median number of image processed}{{min}_{s} per unit time}$$

Similarly, maximum rationalization is the process of the total number of inputs working with the maximum number of images processed to accurately detect a tumor spot concerning unit time. Thus, it is determined as follows,

8 $${max}_{R}=Total number of image \times \frac{maximum number of image processed}{{max}_{s} per unit time}$$

When the processing of an image is done based on the availability of the image and the segmentation of the image based on diagonals for both maximum and minimum rationalization, that is,

9 $$\left|{min}_{R}\right|= \left({\mu }_{(a,b)}\times log\left(\frac{\delta [\left(p\right)to(r)]}{da}\right)\right)$$

Similarly,

$$\left|{max}_{R}\right|= \left({\mu }_{(a,b)}\times log\left(\frac{\delta \left(p\right)}{da}\right)\right)$$

where δ is the number of segmented images and data states the availability of an image; thus, these functions are used for generating max and min rationalizations.

**Figure 5:**
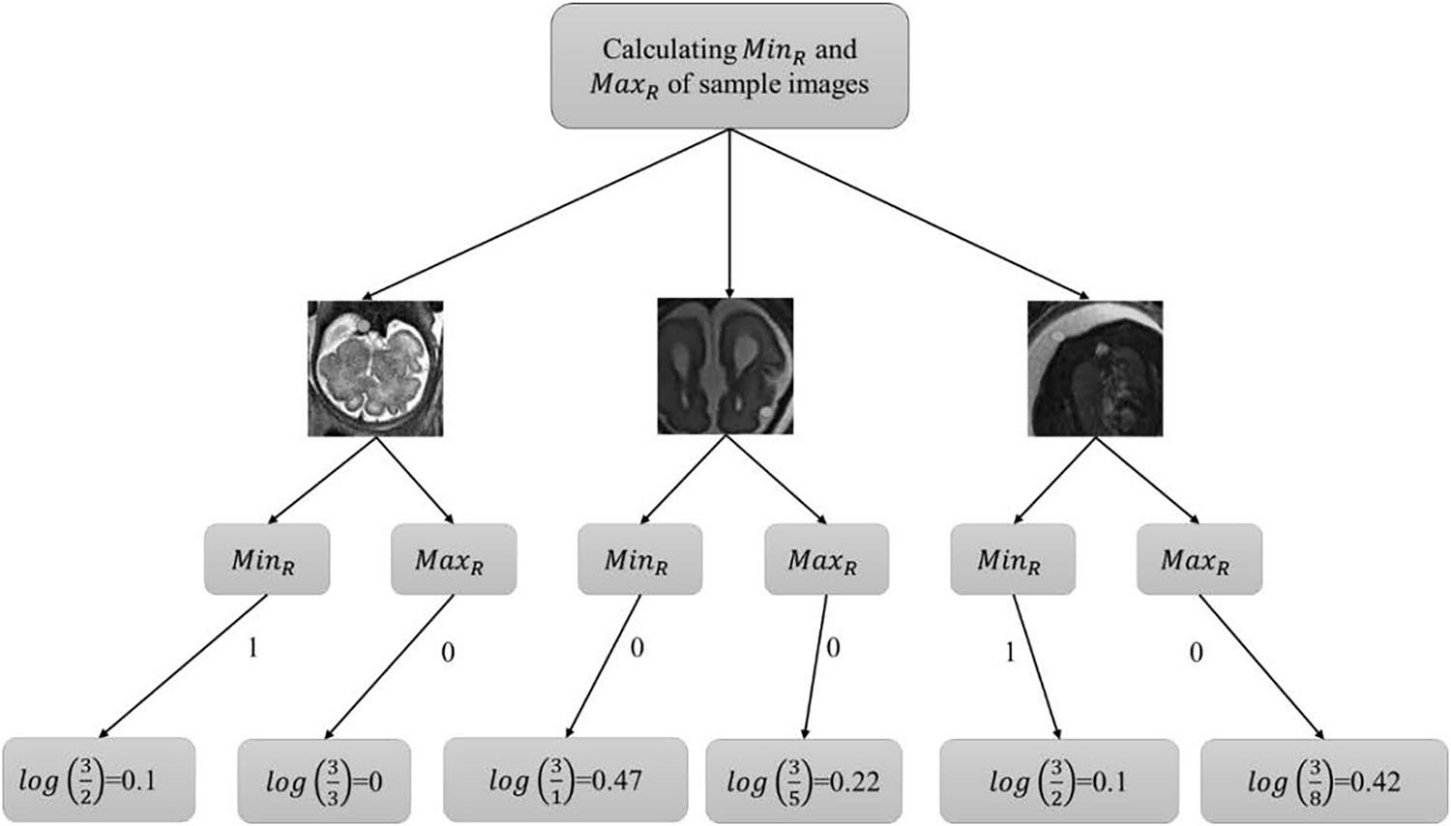
Maximum and minimum rationalization based on image.

Figure 5 discusses maximum and minimum rationalization based on image calculation of the min and max rationalization of images for segmentation with different conditions represented in the matrix form of the max and min rationalization of the picture. The three additional MRI images were considered for calculating the ${min}_{R}$ and ${max}_{R}$. With different conditions 0 and 1 for each image^32^. Let the value returned from step 1 and the availability of the image be represented as ${\alpha }_{\left(a,b\right)}$. The analysis is considered with the acquired three images, then the matrix will be as follows,

10 $${\alpha }_{\left(a,b\right)}\times {\left[1 0 1 1 1 0 1 0 0 0 0 1 10 \right]}^{T}$$

Thus, the availability of images for diagnosis tumor recognition is a minimum rationalization. The matrix is replaced with a minimum value of 0.176 at every position of 1 in the matrix. Then the ${min}_{R}$ And the availability of images is determined as,

11 $$\left|{\alpha }_{\left(a,b\right)}\times loglog \left(\frac{\vartheta (p)}{da}\right) \right|$$

12 $$\left|{\alpha }_{\left(a,b\right)}\times loglog \left(\frac{\vartheta (p)}{da}\right) \right|\equiv \left[1 0 0 1 \right]=0.03097$$

where, 1 = 0.176; $$\left[1 0\right]$$ = 0.477

13 $$\left|{\alpha }_{\left(a,b\right)}\times loglog \left(\frac{\vartheta (p)}{da}\right) \right|\equiv \left[1 0 0 1 \right]=-0.08394$$

Then the α_(a,b)_ is extracted from the following result of the matrix.

14 $$\left|{\alpha }_{\left(a,b\right)}\times loglog \left(\frac{\vartheta (p)}{da}\right) \right|\equiv \left[1 0 0 1 \right]=0.05297$$

Thus, from the above matrix, it is determined that row and column 1 provide the positive result based on ${min}_{R}$ With the value of higher rationalization of image for diagnosis determination.

#### Vulnerability scanning and threat assessment

**Figure 6:**
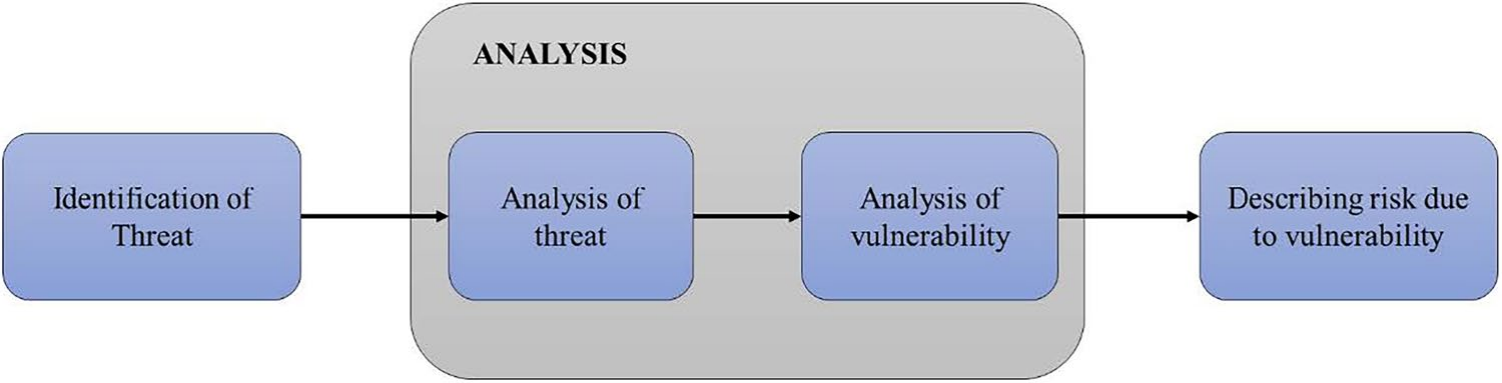
Risk assessment process by analyzing threat and vulnerability.

The identified threat in IDS is evaluated to determine the threat type and the network's vulnerability. Thus, the effects of the threat are identified using the vulnerability scanning process; therefore, it is also known as vulnerability assessment which evaluates systems, networks, and computers for finding cyber threat weaknesses or vulnerabilities^33^. After identifying the threat, it is imperative to know the details about it to provide an effective solution to reduce the exposure generated by the threat. There are 4 steps for analyzing the threat to reduce the network vulnerability (Fig. 6).

#### Vulnerability normal data scanning

In the network, standard data is used to analyze continuous data. The shared data is the simple process of communication that does not produce higher risk; for example, establishing a connection for the communication process is the average data. As the packets are transmitted through the network, some packages might be affected due to less security as these data don’t contain sensitive information. Thus, to find the affected or abnormal data from standard data, each continuous data is observed and evaluated to find the ratio or attribute of the defect. The length of serial data is N, so the scanning process takes place N-1 times to find the characteristics of the fault. In the beginning, the level of an attribute is null or 0. Then after considering the 1st observation for analysis, the attribute level turns 1 and continues to increase. The standard distribution method allocates each attribute within the observed area when considering the invalid state. The ratio of damage for individual observations is determined based on alternative lemmas^34^. That is, the statement might be increased or decreased. When the attribute is found to be an increased value, the observed data is within the range; otherwise, it is determined outside the field. Thus, before deciding the number of observations, the scanning process collects the information by placing the vulnerability scanner in the network to find the possibility of insufficient data threatening the network or endpoints. A vulnerability scanner is a tool for automatically testing a network's vulnerability for defective or malicious standard data that occurred due to a threat. The network vulnerability is evaluated based on the test of the likelihood log ratio. Thus, it is associated with the observed data at a maximum of 70% defective. The likelihood of network vulnerability is higher. When the balance of insufficient data is minimum, then the exposure of the network is less^35^.

In the network, each standard and abnormal data is observed and evaluated to find the ratio or attribute of the defect. The length of continuous information is N, so the scanning process takes place N-1 times to find the characteristics of the fault. In the beginning, the level of an attribute is null or 0. Then, after considering the 1st observation for analysis, the attribute level turns one and continues to increase. The standard distribution method allocates each attribute within the observed area when considering the invalid state.

**Figure:**
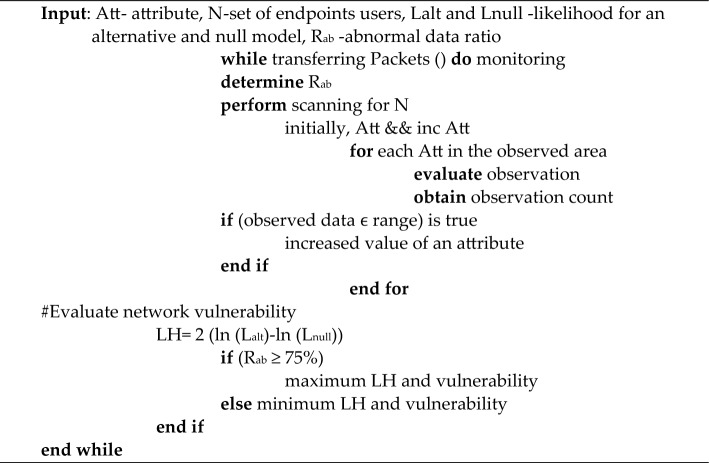
Algorithm: Algorithm for vulnerability normal data scanning.

The defective or malicious average data that occurred due to a threat. The vulnerability scanner collects data in the network to find the possibility of insufficient data threatening the network or endpoints. The network vulnerability is evaluated based on the test of the likelihood log ratio. The likelihood of network vulnerability is higher, and when the balance of defective data is minimum, then the exposure of the network is less. Thus, it is associated with the observed data at a maximum of 70% inadequate.

As a consequence of the aforementioned research paradigm, a groundbreaking method for nonlinear dynamics analysis leveraging visibility graph techniques was formulated. Here in our research paper mentioned above we explained how to segment and classify glioblastoma brain tumors on MRI in an adult. We carried out this research using MRI scan images taken when the baby was in the womb and we used that image to perform Fetus brain tumor extraction. We took the research explanations for this through research paper^36–40^ and from them we gave our research conclusion.

## Results and discussion

The massive growth of abnormal cells in the brain causes brain tumor glioblastoma, which might be dangerous to humans diagnosed and identified by brain MRI images. In this proposed system, a MATLAB tool is used to analyze brain tumor glioblastomas using a Micro Dicom database consisting The MRI images with 256 × 256 with the intensity of images obtained from the Kaggle dataset https://www.kaggle.com/datasets/navoneel/brain-mri-images-for-brain-tumor-detection. This dataset contains the 1690 MRI scans of the training and testing data set. It has 958 images of tumor data and the other 732 images of healthy brain data^41^.

The proposed hybrid TAE performs the brain tumor glioblastoma segmentation on the Kaggle testing dataset. PIS is evaluated by the MATLAB tool. The image segmentation for 200 images. For training, 120 photos were taken, 66 for tumor images, and standard brain images were 24. 10 testing images, the 6 images with tumors and 4 without tumors, as shown in Table 1. The cross-validation method is integrated into our proposed process, which can improve the accuracy of the brain tumor glioblastoma classification. The calculation of mean square error, peak signal-to-noise ratio, structural similarity ratio, and dice coefficient is performed as shown in Table 2. These tests are approximately 85% efficient for the segmentation of brain tumor glioblastomas.

**Table 1:** Preparation for training and testing brain images.

Image count	Training data (120)		Testing data (80)	
Total	Normal	Tumor image	Normal	Tumor image
200	54	66	16	64

**Table 2:** Comparison of methods through the performance metrics.

Techniques	MSE	PSNR	SSIM	Average dice score
GA [Ouseph et al.]	0.34	0.76	0.861	0.79
KNN [Kharrat et al.]	0.25	0.78	0.941	0.82
K-SVM [Mandle, A. K]	0.022	0.81	0.926	0.84
TAE-PIS	0.0346	0.89	0.985	0.95

As highlighted in the tables and figures, our proposed TAE-PIS method outperforms existing approaches in terms of Mean Squared Error (MSE), Peak Signal-to-Noise Ratio (PSNR), and Structural Similarity Index (SSIM). The TAE-PIS method demonstrates higher accuracy and efficiency in segmenting brain tumor glioblastomas from MRI images compared to traditional methods such as Genetic algorithms (GA), K-nearest neighborhood (KNN), and kernel-based support vector machine (K-SVM).

The novelty of our approach lies in its integration of Time Attribute Extraction (TAE) and Partial Image Segmentation (PIS), which enhances the accuracy and reliability of brain tumor segmentation. By achieving higher performance metrics and demonstrating superior results in comparison with existing techniques, our research contributes significantly to advancing the field of medical image analysis.

### Mean squared error (MSE)

The MSE (mean squared error) is defined as squaring the discrete principles or average of the number of squares of the defects, which is calculated by subtracting the input image segmented. MSE is listed below,

15 $${\text{MSE}}=\frac{1}{m\times n}*{\left(I\left(x,y\right)-S\left(x,y\right)\right)}^{2} x\epsilon \left[0-m-1\right], b\epsilon [0-n-1]$$

MRI brain image input is m × n format. I (x, y) refer to the input brain image and S (x, y) refers to the segmented brain image. When the MSE value of the brain image segmentation is tiny, we might get a better PSNR value.

**Figure 7:**
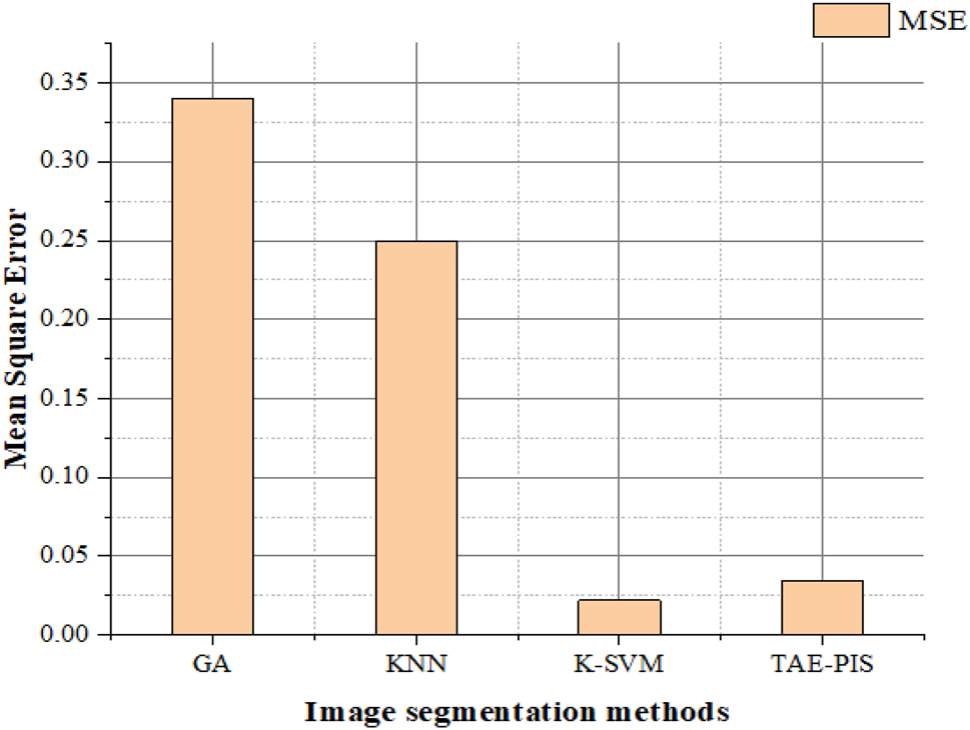
Comparison of MSE in image segmentation methods.

Our proposed TAE-PIS image segmentation method is compared with the above approaches, such as Genetic algorithms (GA), K-nearest neighborhood (KNN), and kernel-based support vector machine (K-SVM). The mean square error value of various image segmentation methods is shown in Fig. 7. The graph shows that the TAE-OIS method has a smaller MSE value than the other method, so the TAE-PIS method is better for image segmentation. When the MSE value is higher, the PSNR value is lower and the system performance remains poor, the MSE value is lower than the PSNR value is higher and the system performance remains better.

### PSNR (peak signal-to-noise ratio)

PSNR values in the range of 40 to 100 dB are less susceptible to noise. The PSNR generally shows a noise immunity of brain images which depends on the MSE value. When the PSNR value is higher than the MRI brain image of noise is low. The most considerable pixel value in the input MR brain image is designated as a maxi.

16 $${\text{PSNR}}= 10\mathrm{ log}\left(\frac{{Max}^{2}}{MSE}\right)$$

**Figure 8:**
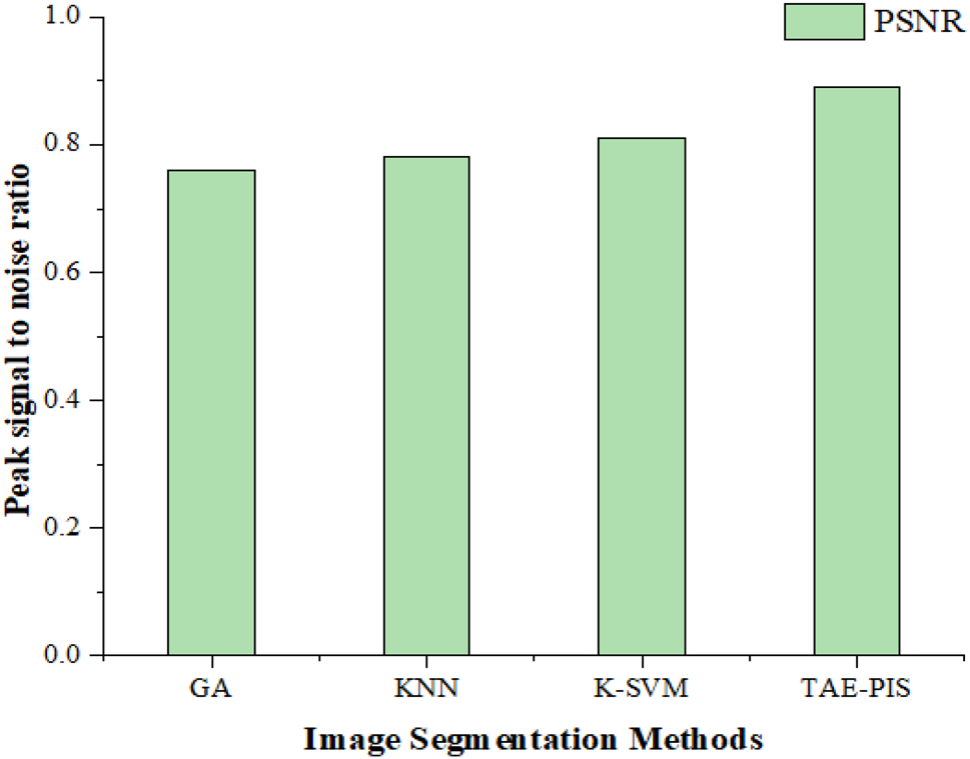
Comparison of PSNR in image segmentation methods.

Image segmentation is the first stage of identifying the exact brain tumor glioblastoma spot. According to Fig. 8, various image segmentation methods are compared with our proposed method, Segmentation methods that contain the highest PSNR value are chosen as the better image segmentation method and it is also more suitable for segmenting the image. The time attribute extraction and partial image segmentation method (TAE-PIS) are applied to the MRI brain images, and different peak signal-to-noise values are determined. From the above statement and according to Fig. 8, the TAE–PIS method is 8% higher than the K-SVM method, 11% higher than the KNN method, and 13% higher than the GA method. Finally, the TAE–PIS is better than the other methods.

### Structural similarity index (SSIM)

The Structural Similarity Index (SSIM) shows when the quality of the brain image is low due to data compression, data transmission losses, or another image processing approach. SSIM may be described as,

17 $${\text{SSIM}}=\frac{2{\delta }_{x}{\delta }_{y}}{{({\delta }_{x})}^{2}+{({\delta }_{y})}^{2}+{C}_{2}}*\frac{2xy}{{x{^{\prime}}}^{2}+{{y{^{\prime}}}^{2}+C}_{1}}+\frac{{\delta }_{xy}}{{\delta }_{x}{\delta }_{y}}$$

An advanced SSIM value means better luminance, contrast, and structural material preservation.

**Figure 9:**
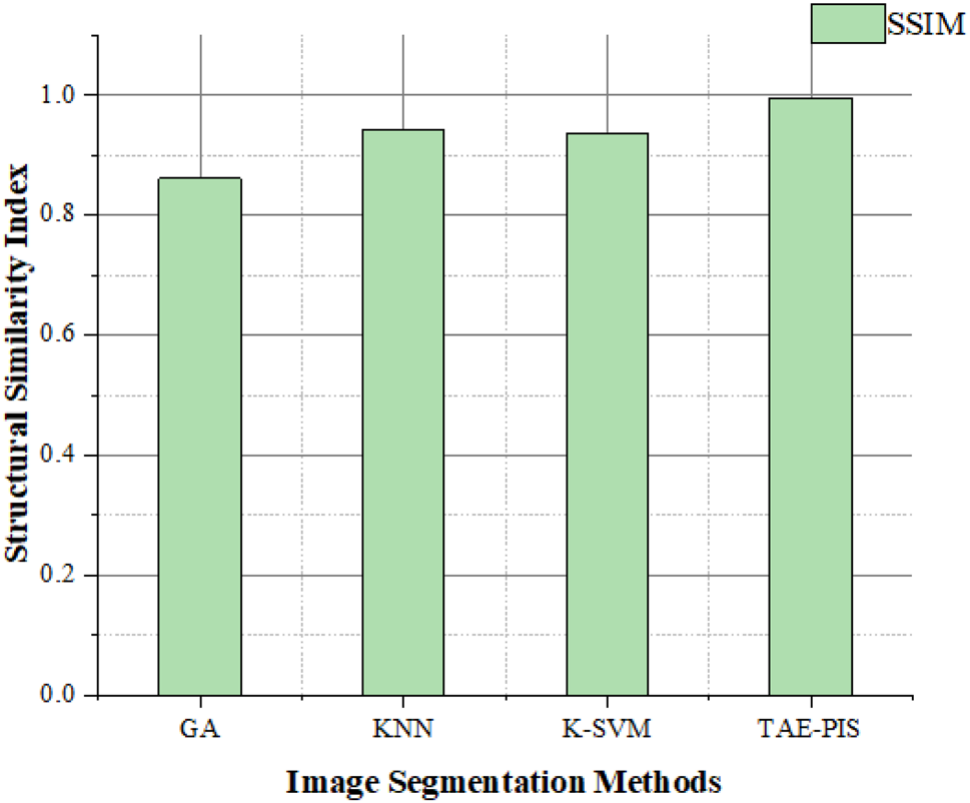
Comparison of SSI in image segmentation methods.

The relationship between the SSIM index and image segmentation methods is shown in Fig. 3. Figure 9 clearly shows the structural similarity index value of each image segmentation method comparison. By using the GA method for image segmentation, it has 86% similarity with the input image. The KNN method has a 94% similarity with a given input image. The K-SVM method has a 92% similarity with the input image. Finally, the TAE–PIS method has a parallel with the given input image is 98%, which means the structural similarity index of this method is comparatively higher and better than other image segmentation methods.

### Dice coefficient

**Figure 10:**
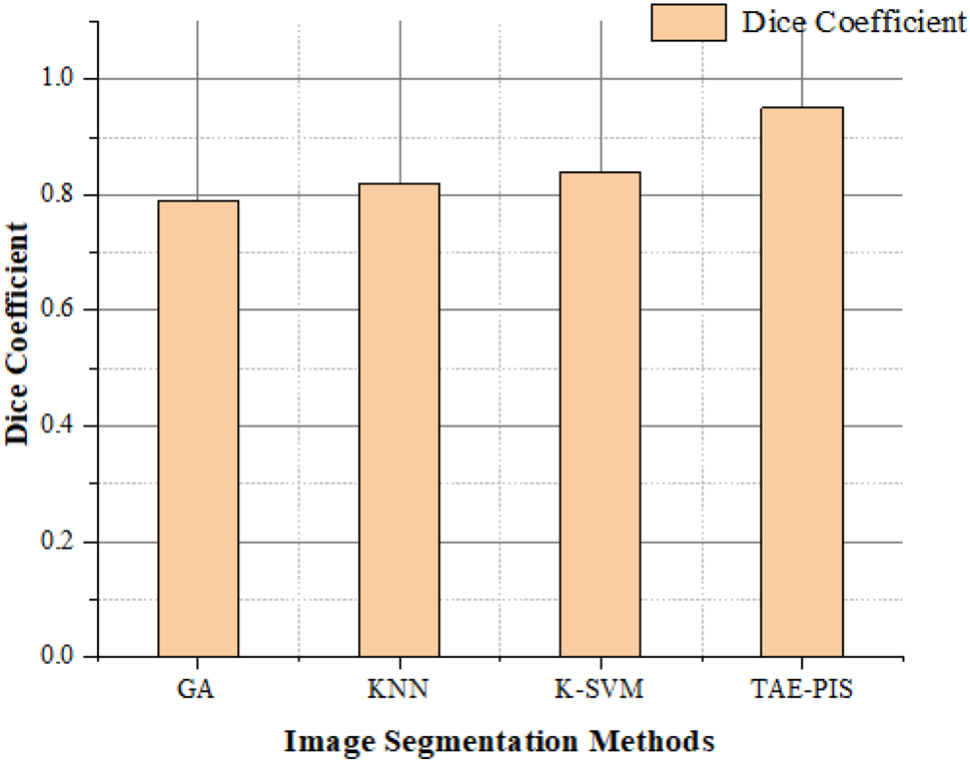
Comparison of dice co-efficient in image segmentation methods.

The dice coefficient is referred to as an indicator for comparing the two brain images, and it is known as the dice similarity index. It is written as,

18 $$\mathrm{DICE }= 2*\frac{\left|T\bigwedge G\right|}{\left|T\right|+\left|G\right|}$$

where T ∈ {0, 1} shows the tumor area determined by the hybrid method and G ∈ {0, 1} shows the ground truth as determined by experts. The dice coefficient has a minimum (0) and maximum (1) value. When the dice coefficient is higher, it shows that two brain images overlap more. The tumor segmentation was divided into four factors,

The segmentation technique for Brain tumor glioblastoma MR images based on the TAE-PIS method was proposed. Figure 10 shows the dice coefficient value of various image segmentation methods with our proposed method. The dice coefficient value of GA is 0.74, KNN is 0.82, K-SVM is 0.84, and TAE-PIS has a 0.95 value of 95%. Therefore, when the brain image segmentation dice coefficient is higher, it is better than the other Brain tumor glioblastoma segmentation methods. From this, the TAE–PIS method is better than the other method.

Table 3 comprises the brain tumor glioblastoma approximation and Table 4 shows the metrics for analyzing the performance of the image segmentation methods. Table 4 shows the compassion of accuracies for increasing the count of MRI brain images. The True positive rate (TPR) measures the proportion of the brain tumor glioblastoma images correctly identified as positive by the proposed method. It is also called sensitivity which is estimated based on the confusion matrix as follows,

19 $${\text{TPR}} = {\text{TP}}/{\text{TP}} + {\text{FN}}$$

**Table 3:** Brain tumor glioblastoma image segmentation is based on the below approximation.

Estimation		
Input brain MRI image	True positive (TP)	When the tumor was correctly identified
	False positive (FP)	When the tumor was incorrectly identified as a standard brain image
	True negative (TN)	When the standard brain image was correctly identified
	False negative (FN)	When a standard image was incorrectly identified as a tumor image

**Table 4:** The compassion of accuracies for brain images.

Image count	Actual positive rate (%)	Valid negative rate (%)	Precision (%)	Accuracy (%)
10	74	80	48	50
20	72	74	52	54
30	87	67	57	59
40	87	50	63	64
50	90	62	59	72
60	92	50	64	77
70	95	46	68	80
80	97	52	65	85

The True negative rate (TNR) measures the proportion of the standard brain images correctly identified as unfavorable by the proposed method. It is also called specificity, which is estimated based on the confusion matrix as follows,

20 $${\text{TNR}} = {\text{TN}}/{\text{TP}} + {\text{FN}}$$

**Figure 11:**
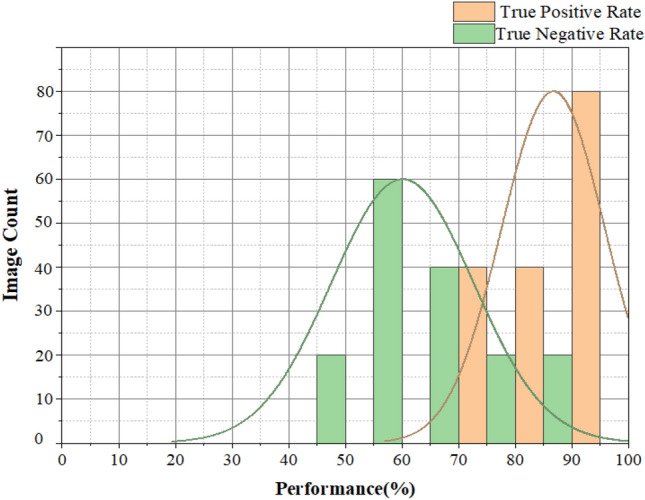
Actual Positive rate and Negative valid rate for brain image.

Figure 11 presents the proper positive and negative rates for an increasing count of input MRI brain images. The actual positive and true negative rate methods are important factors for analyzing the proposed method's performance. This proposed method can correctly identify a brain tumor glioblastoma image and a standard brain image.

**Figure 12:**
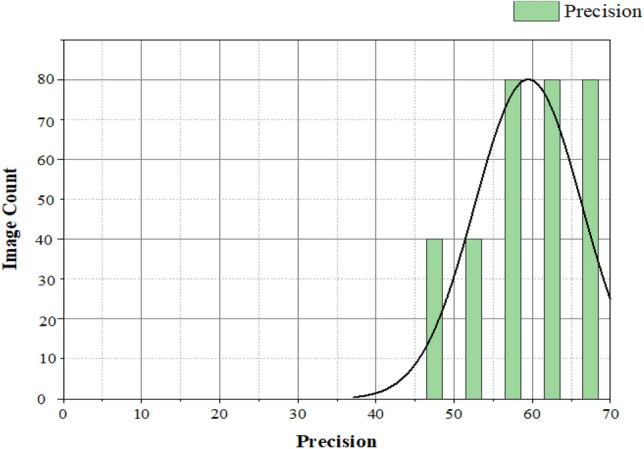
Precision for brain image.

The precision of the proposed method is seen in Fig. 12. the accuracy is estimated as follows,

21 $${\text{Precision P }} = {\text{TP}}/{\text{TP}} + {\text{FP}}$$

This proposed method can correctly identify a brain tumor glioblastoma image and a standard brain image.

The accuracy is the overall count of correctly segmented brain tumor glioblastoma images, which is calculated as follows,

22 $${\text{Accuracy}}:{\text{ A}} = {\text{TP}} + {\text{TN}}/{\text{TP}} + {\text{FP}} + {\text{TN}} + {\text{FN}}$$

**Figure 13:**
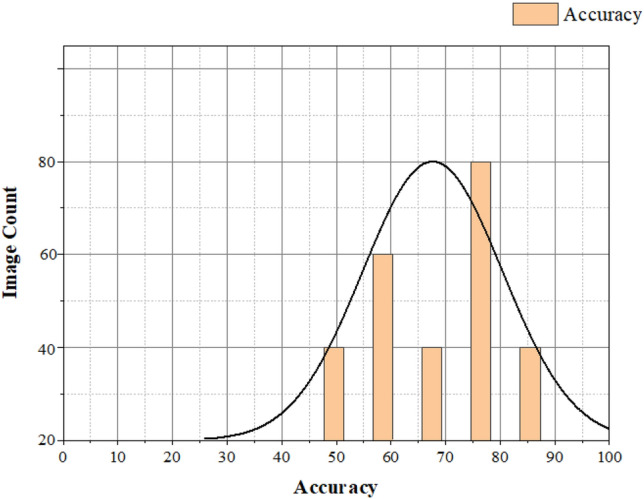
Accuracy for the image segmentation with increasing count of brain images.

In Fig. 13, the count of brain images and accuracy will be plotted on the Y and X axes, respectively. By increasing the count of brain images, the proposed method can effectively identify and segment the brain image. We have already estimated the proposed method performance metrics. We then compared the proposed method with various brain tumor glioblastoma image segmentation and identification methods shown in Table 5. The different techniques are Genetic algorithm (GA), Convolution neural network (CNN), fuzzy-based minimum, and maximum neural network (Fuzzy min–max NN).

**Table 5:** Comparative performance metrics analysis with various methods.

Methods	Performance metrics		
	True positive rate (%)	True negative rate (%)	Accuracy (%)
GA	80.52	77.23	80.82
CNN	83.21	79.01	82.13
Fuzzy min–max NN	85.34	80.34	85.61
K-SVM	87.82	82.71	87.03
TAE-PIS	98.34	97.42	98.12

**Figure 14:**
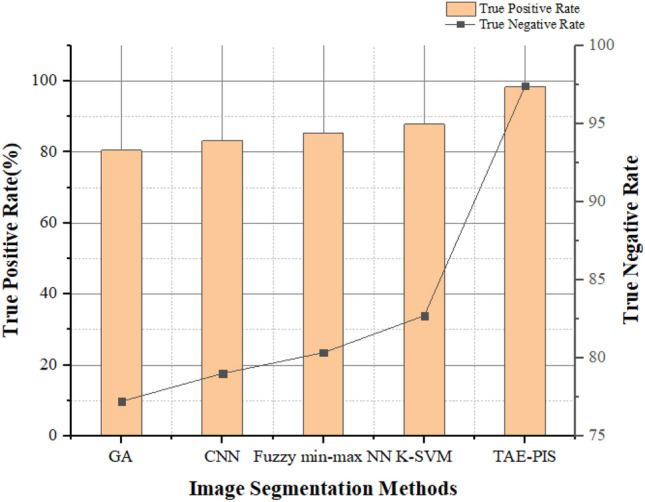
True positive rate and true negative rate for comparative analysis.

Figure 14 demonstrates the true positive rate and true negative rate of various image segmentation methods. The TAE–PIS method has effectively segmented the brain tumor glioblastoma image as positive and normal brain image as negative by comparing the other methods. The GA has 80.52% and 77.23%, CNN has 83.21%and 79.01%, fuzzy min–max NN has 85.34% and 80.34%, and K-SVM has 87.82% and 82.71% of true positive rate and true negative rate respectively. The TAE-PIS method has a true positive rate of 98.34% and a true negative rate of 97.42 which is higher than other methods. Finally, the TAE-PIS method is better than other methods.

**Figure 15:**
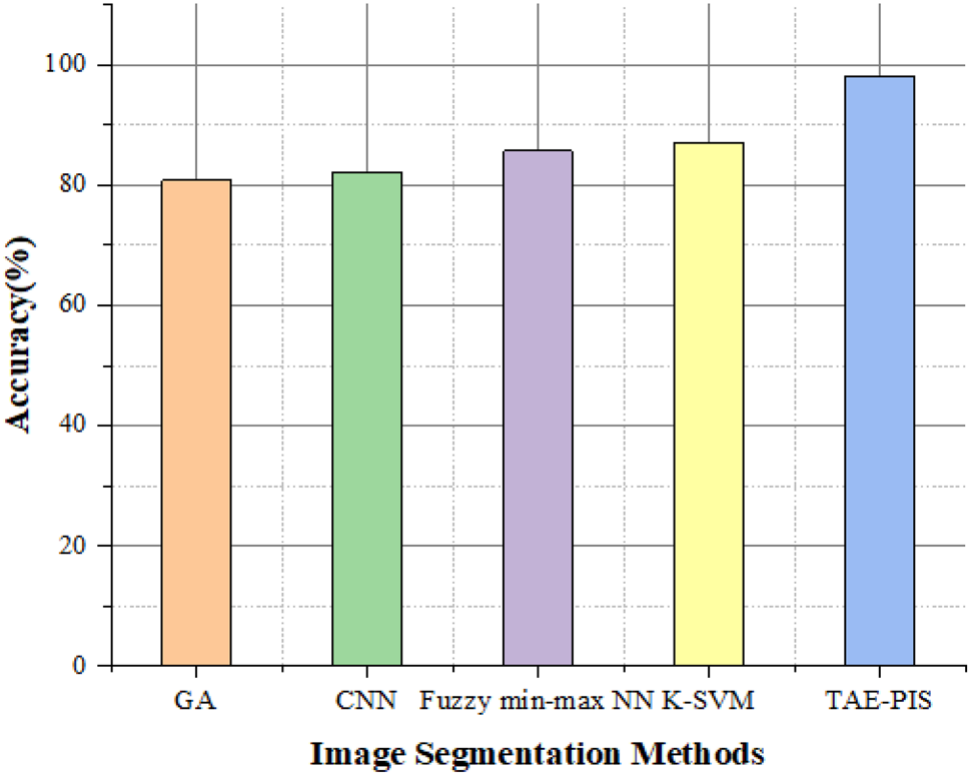
Comparison of accuracy values of the proposed method with GA, CNN K-SVM.

Figure 15 compares the accuracy values of the proposed method with GA, CNN K-SVM, and fuzzy min–max NN methods. From the above Fig. 15, the proposed image segmentation method performs better than other methods in terms of accuracy. The proposed method has 98.12% accuracy and 11.3%, 12.51%, 15.99%, and 17.3% better than the Genetic algorithm (GA), Convolution neural network (CNN), fuzzy-based minimum and maximum neural network (Fuzzy min–max NN) and kernel-based support vector machine respectively. From this estimation, our proposed method has higher accuracy than the other image segmentation methods. After brain image segmentation, the octagon structure is established for image recognition and diagnosis to the exact brain tumor glioblastoma spot from the rain images. The octagon method can split the brain image until all un-spotted regions of an image get removed and find the brain tumor glioblastoma spot. Rationalization is reducing the diagonals of an image from a larger dimension to lower dimensions to recognize diagnosis tumors from an input image. Thus, maximum and minimum rationalization of ideas helps generate a different state of equilibrium for extracting an output as diagnosis tumor detection. Table 6 shows the possibility of finding the brain tumor glioblastoma in the MRI brain image before and after applying the maximum and minimum rationalization with the octogen structural methods.

**Table 6:** Comparison of brain tumor glioblastoma spot identification before and after applying the rationalization.

Iteration count	Before applying maximum and minimum rationalization			After applying maximum and minimum rationalization		
	Accuracy (%)	Identification error	Response time	Accuracy (%)	Segmentation error	Response time
1	56	2.37	0.65	68	1.62	0.51
2	63	3.96	0.54	70	3.45	0.46
3	50	4.28	0.72	59	0.87	0.22
4	64	2.25	0.70	72	2.26	0.62
5	59	9.05	0.72	65	7.56	0.68
6	70	4.54	0.74	74	3.21	0.32
7	78	4.23	0.75	82	3.21	0.56
8	72	5.56	0.76	78	4.32	0.57
9	80	6.50	0.77	89	4.90	0.64
10	85	4.34	0.78	92	3.21	0.72

**Figure 16:**
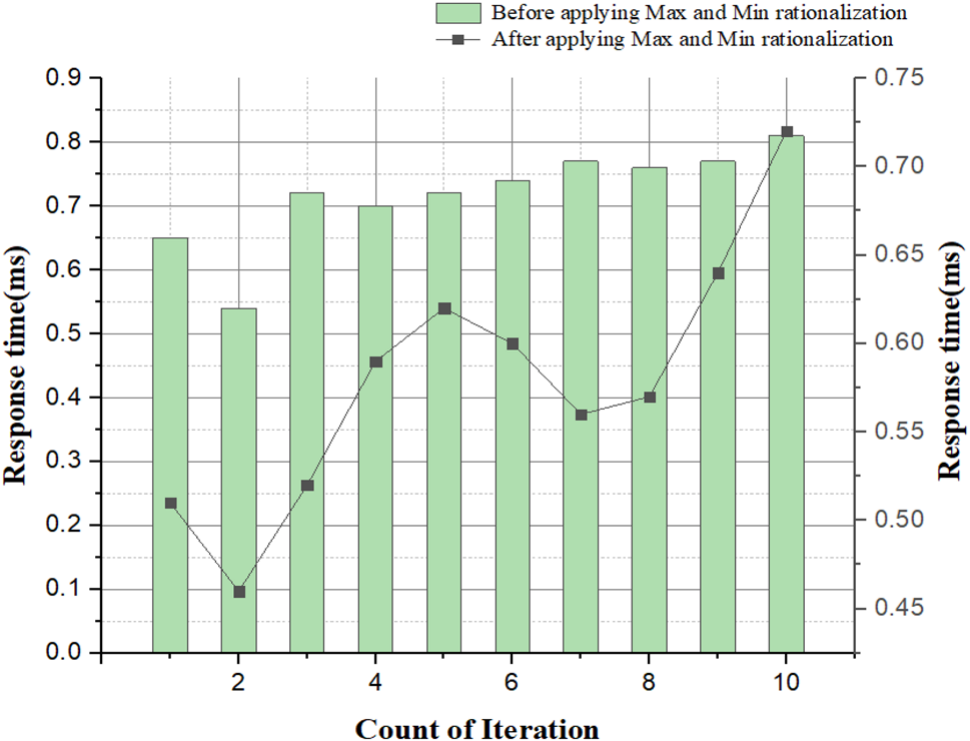
Response time vs. no. of iterations.

Figure 16 shows the image identification time during the increasing count of brain images before and after applying the minimum and maximum rationalization methods. Response time is the time taken for the process to complete. From the graph, we can see that, before applying the maximum and minimum rationalization methods, the proposed techniques for tumor spot identification gives approximated results of the exact tumor-diagnosis. Still, after using the maximum and minimum rationalization methods, the proposed system produces an actual tumor-diagnosis location from the brain image. The proposed system with maximum and minimum rationalization methods takes a lesser response time than the one without maximum and minimum rationalization methods. So, applying these methods gives a better result regarding quick responses for brain tumor glioblastoma identification.

**Figure 17:**
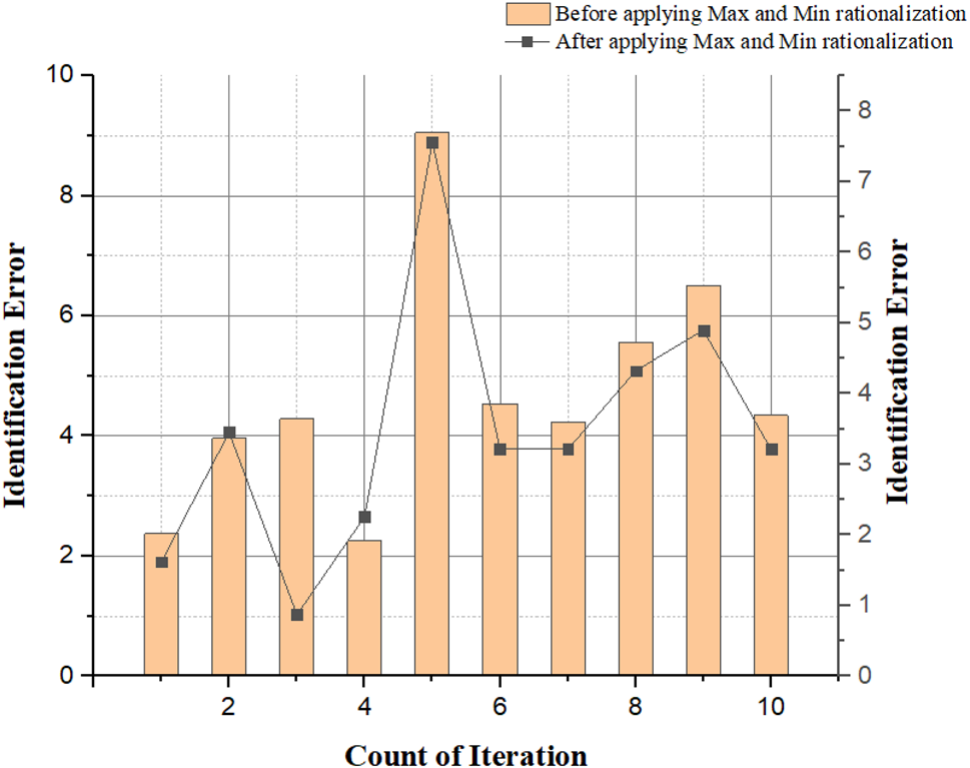
Identification error vs. image segmentation iteration.

Figure 17 shows the image identification error during the increasing count of brain images before and after applying the minimum and maximum rationalization methods. From the graph, we can see that, before using the maximum and minimum rationalization methods, the proposed techniques for tumor spot identification gives approximated results of the exact tumor-diagnosis. Still, after applying the maximum and minimum rationalization methods, the proposed system produces an actual tumor-diagnosis location from the brain image. During this process, an identification error may occur. Still, a proposed design with maximum and minimum rationalization methods has lesser error than the proposed system without maximum and minimum rationalization methods. So, applying these methods gives a better result in terms of reduction in error.

**Figure 18:**
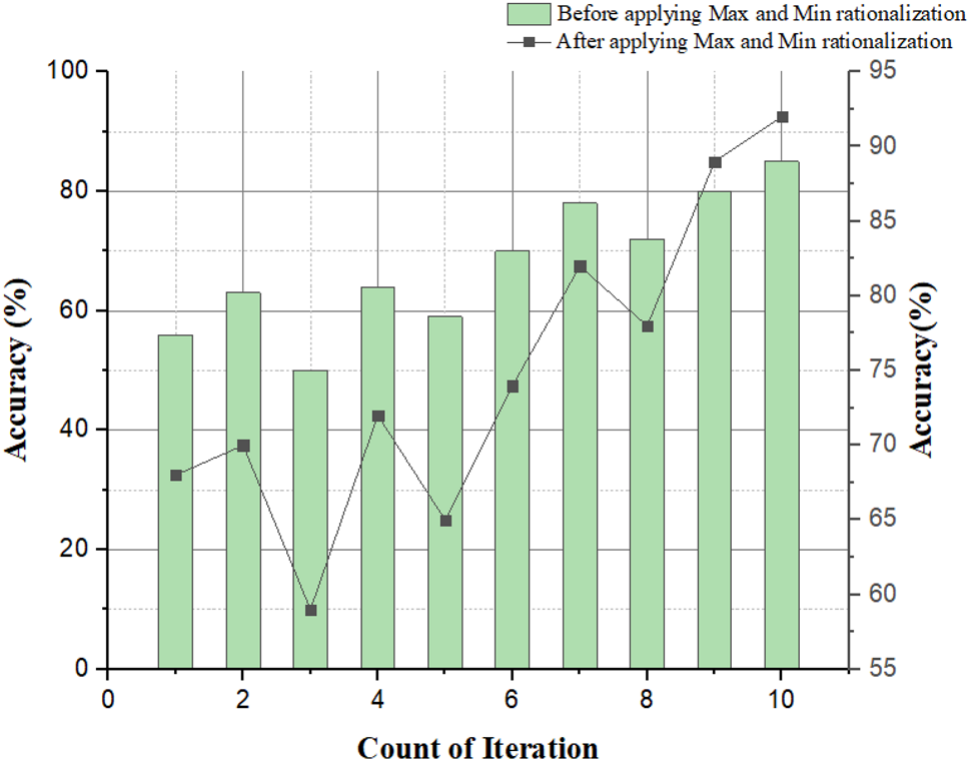
Accuracy vs. no. of iteration.

Figure 18 depicts the accuracy graph. The vertical axis is the accuracy that represents the before and after applying the minimum and maximum realization for the image segmentation process based on the increasing count of iterations. The horizontal axis represents the growing counts of iteration. In our proposed system, before applying minimum and maximum rationalization, we only get an approximate spot of a brain tumor glioblastoma in the brain image. Still, we might get an accurate diagnosis after using this minimum and complete rationalization method. Based on these results, we can say the accuracy after applying min and max rationalization methods in the system are comparatively higher than before using these methods for image segmentation.

The MRI brain image might contain a very small or micro-level tumor. Considering this state, a division of the image based on time division with the geometric shape is used to recognize the image. The whole brain image is regarded as N, then let the tumor's diameter be tiny. The area of the brain tumor glioblastoma is determined by enumerating the count of tumor pixels. And then, the location of the normal brain portion is determined by computing the count of normal brain pixels. The brain tumor glioblastoma ratio is calculated as follows,

23 $${\text{Ratio}}=\frac{{A}_{Tumor}}{{A}_{normal brain}}*100\%$$

The calculation of average error value of the brain tumor glioblastoma area and normal brain area is computed as follows,

24 $${\text{E}}= \frac{Mentioned tumor value-Estimated tumor value }{Mentioned tumor value}$$

The error testing of tumor extent is used to test how effective the identification of brain tumor glioblastomas has been. Table 7 shows the average error in identifying the brain tumor glioblastoma area.

**Table 7:** An average error in the identification of brain tumor glioblastoma area.

Iteration count	Ratio = $\frac{tumor area}{Brain Area}*100$ (%)	Mentioned value of tumor (mm^2^)	An estimated value for tumor (mm^2^)	Error (%)
1	366	346,828	319,823	7
2	56	28,068	24,723	11
3	243	78,055	74,092	5
4	5	11,893	10,623	10
5	76	12,725	11,432	10
6	25	78,055	73,232	6
7	83	68,289	65,234	4
8	385	11,277	10,273	9
9	213	2584	2413	7
10	72	1445	1173	18

**Figure 19:**
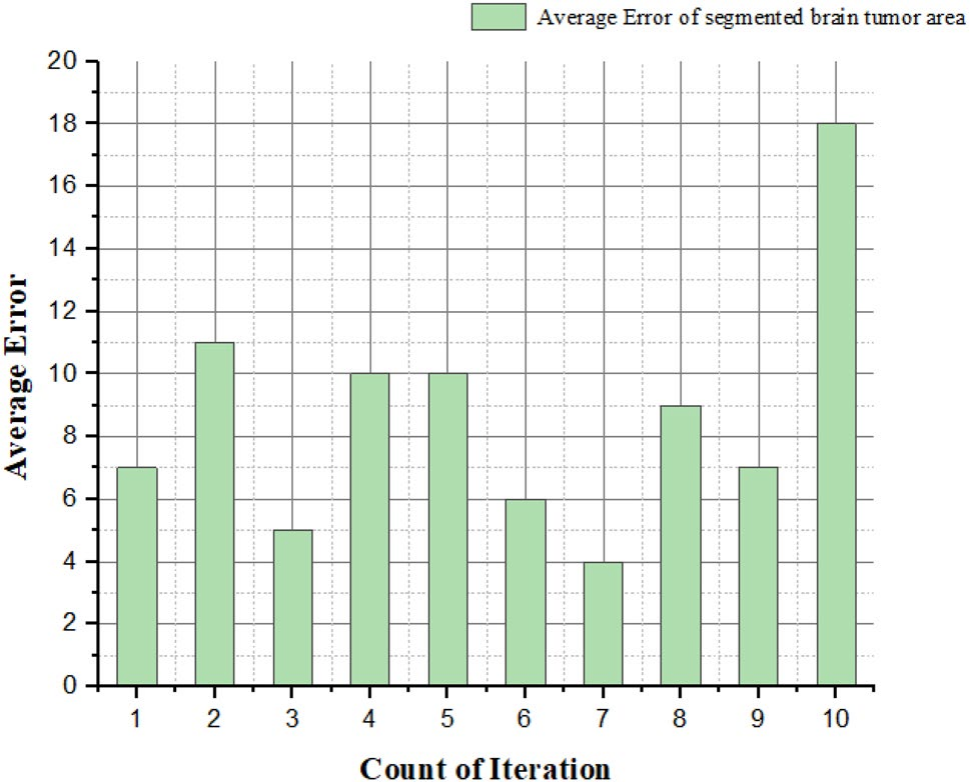
An average error of a brain tumor glioblastoma area in brain image.

Figure 19 shows the average error during the brain tumor glioblastoma identification by increasing the count of brain image iteration using these methods. Compared to other methods, our proposed system has fewer errors for segmenting and identifying the brain tumor glioblastoma spot. Keeping in mind that our research will help the medical field if we find caustic diagnostic equipment for high-grade fetal brain tumors, and as you said, the average patient with glioblastoma of the brain is incurable for about 1.5–2 years. We propose this research with the intention that our concept may be useful in the future by conceptually leaning on the processes of invented equipment.

## Conclusion

In this study, we employed enhanced division time attribute extraction and max- and min-rationalizing techniques for image analysis. Specifically, we applied these methods to identify glioblastoma, a type of brain tumor, in MRI images, aiming to enhance treatment effectiveness. Our emphasis on machine image processing underscores its significance in understanding fetal development, with potential implications for brain diagnosis in adults using MRI images in future research. We are committed to addressing limitations, incorporating diverse datasets, and integrating advanced techniques to contribute to the field of brain tumor detection and diagnosis.

To support our findings, we referenced various research articles focusing on brain tumor detection and classification, highlighting the role of engineered biomimetic nanoparticles in targeted therapy against glioblastomas. Notably, our utilization of Self-Organizing Maps (SOMs) for verification resulted in significant improvements, achieving an accuracy of 96.5% and reducing response times to 7.5 seconds. Furthermore, our adaptation of image segmentation techniques resulted in a significant enhancement in accuracy to 98.12%, further demonstrating the effectiveness of our approach. Looking ahead, we plan to extend our research to brain diagnosis in adults, leveraging MRI images for comprehensive analysis. Our commitment to advancing this field of medicine underscores our dedication to improving diagnostic accuracy and treatment outcomes for patients.

As we move forward, one promising direction for future research is the exploration of deep learning techniques for brain tumor diagnosis. By leveraging convolutional neural networks (CNNs) and recurrent neural networks (RNNs), we aim to develop more sophisticated models capable of detecting subtle patterns in MRI images with higher accuracy and efficiency. Additionally, incorporating multi-modal data fusion approaches, such as combining MRI with other imaging modalities or clinical data, holds promise for enhancing diagnostic capabilities and advancing personalized medicine in neuro-oncology. Through these efforts, we anticipate further advancements in brain tumor diagnosis and treatment, ultimately benefiting patients worldwide.

## Data Availability

The datasets used during the current study are available from the corresponding author on reasonable request.

## References

[1] Malkki, H. Proteomic profiling could facilitate glioblastoma diagnosis. *Nat. Rev. Neurol.* **10**, 484. 10.1038/nrneurol.2014.142 (2014). 25092416 10.1038/nrneurol.2014.142

[2] Gelardi, E. L. M. *et al.* Curcumin-based-fluorescent probes targeting ALDH1A3 as a promising tool for glioblastoma precision surgery and early diagnosis. *Commun. Biol.* **5**, 895. 10.1038/s42003-022-03834-7 (2022). 36050388 10.1038/s42003-022-03834-7 PMC9437101

[3] Wang, J. *et al.* Clonal evolution of glioblastoma under therapy. *Nat. Genet.* **48**, 768–776. 10.1038/ng.3590 (2016). 27270107 10.1038/ng.3590 PMC5627776

[4] Mandle, A. K., Sahu, S. P. & Gupta, G. Brain tumor glioblastoma segmentation and classification in MRI using clustering and Kernel-based SVM. *Biomed. Pharmacol. J.* **15**(2), 699–716 (2022).

[5] Ouseph, N. C. & Shruti, K. A reliable method for brain tumor glioblastoma detection using cnn technique. *IOSR J. Electr. Electron. Eng.* **1**, 64–68 (2022).

[6] Kharrat, A., Gasmi, K., Messaoud, M. B., Benamrane, N. & Abid, M. A hybrid approach for automatic classification of brain MRI using genetic algorithm and support vector machine. *Leonardo J. Sci.* **17**(1), 71–82 (2010).

[7] Das, S. *et al.* An artificial intelligence framework and its bias for brain tumor glioblastoma segmentation: A narrative review. *Comput. Biol. Med.* **2022**, 105273 (2022). 10.1016/j.compbiomed.2022.105273 35228172

[8] Nehra, M. *et al.* Nanobiotechnology-assisted therapies to manage brain cancer in Childsalized manner. *J. Control. Release* **338**, 224–243 (2021). 34418523 10.1016/j.jconrel.2021.08.027

[9] Mormann, H., Hasse, R. & Arnold, N. Organizing values: The principles of rationalization and individualization. in* Research Handbook on the Sociology of Organizations*, 528–546. (Edward Elgar Publishing, 2022).

[10] Ahanger, I. A. *et al.* Rationalizing the role of monosodium glutamate in the protein aggregation through biophysical approaches: Potential impact on neurodegeneration. *Front. Neurosci.* **15**, 636454 (2021). 33746704 10.3389/fnins.2021.636454 PMC7969894

[11] Lu, J. *et al.* Characterization of immune-related genes and immune infiltration features for early diagnosis, prognosis and recognition of immunosuppression in sepsis. *Int. Immunopharmacol.* **107**, 108650 (2022). 35272172 10.1016/j.intimp.2022.108650

[12] Fang, S. *et al*. Facial image classification of mouse embryos for the animal model study of fetal alcohol syndrome. in *Proceedings of the 2009 ACM symposium on Applied Computing (SAC '09). Association for Computing Machinery*, 852–856 (2009). 10.1145/1529282.1529463. 10.1901/jaba.2009.2009-852 PMC2874915 20502627

[13] Villanueva, D., Enriquez, Y. & Capilit, G. L. The impact of the international rice genebank’s (IRG) on rice farming in Bangladesh. *CABI Agric. Biosci.* **3**(1), 1–14 (2022).

[14] Jensen, L. G. *et al.* Correction of multiple-blinking artifacts in photoactivated localization microscopy. *Nat. Methods* **19**, 594–602. 10.1038/s41592-022-01463-w (2022). 35545712 10.1038/s41592-022-01463-w

[15] Zhao, Y. *et al.* Effects of ultrasound-assisted extraction on the structural, functional and antioxidant properties of *Dolichos lablab* L. *Protein. Process Biochem.* **101**, 274–284 (2021).

[16] Khan, Y. F., Kaushik, B., Chowdhary, C. L. & Srivastava, G. Ensemble model for diagnostic classification of Alzheimer’s disease based on brain anatomical magnetic resonance imaging. *Diagnostics* **12**(12), 3193 (2022). 36553199 10.3390/diagnostics12123193 PMC9777931

[17] Emad, A. M., Rasheed, D. M., El-Kased, R. F. & El-Kersh, D. M. Antioxidant, Antimicrobial activities and characterization of polyphenol-enriched extract of Egyptian celery (*Apium graveolens* L., Apiaceae). *Molecules* **27**(3), 698 (2022). 35163963 10.3390/molecules27030698 PMC8838201

[18] Maganti Syamala, N. J. ABSA: Computational measurement analysis approach for prognosticated aspect extraction system. *TEM J.* **10**(1), 82–94 (2021).

[19] Jiao, R., Liu, X., Zheng, B., Liang, D. & Zhu, Q. *TAE: A Semi-supervised Controllable Behavior-aware Trajectory Generator and Predictor*. arXiv:2203.01261.

[20] Chowdhary, C. L. *et al.* Past, present and future of gene feature selection for breast cancer classification: A survey. *Int. J. Eng. Syst. Model. Simul.* **13**(2), 140–153 (2022).

[21] Castro-Alatorre, N. C. *et al.* Extraction and microencapsulation of bioactive compounds from muicle (*Justicia spicigera*) and their use in the formulation of functional foods. *Foods* **10**(8), 1747 (2021). 34441525 10.3390/foods10081747 PMC8391918

[22] Dutta, B. *et al.* Deep learning for terahertz image denoising in nondestructive historical document analysis. *Sci. Rep.* **12**, 22554. 10.1038/s41598-022-26957-7 (2022). 36581647 10.1038/s41598-022-26957-7 PMC9800433

[23] Yao, Y. F. *et al.* Research on octagon color and fruit shape recognition based on machine vision. *J. Agric. Sci. Technol.* **23**(11), 110 (2021).

[24] Mukherjee, S., Sarkar, S. & Mukhopadhyay, S. Octagon shell based image steganography for avoiding human visual system with lower computational time. in* 2022 Second International Conference on Advances in Electrical, Computing, Communication and Sustainable Technologies (ICAECT)*, 1–5. (IEEE, 2022).

[25] Zhou, Y., Wang, W., Wang, C., Yang, X., Shi, J., & Wei, S. SAR target recognition and angle estimation by using rotation-mapping network. in *2021 IEEE International Geoscience and Remote Sensing Symposium IGARSS*, 3577–3580. (IEEE, 2021).

[26] Jia, H., Yin, Q. & Lu, M. Blind-noise image denoising with block-matching domain transformation filtering and improved guided filtering. *Sci. Rep.* **12**, 16195. 10.1038/s41598-022-20578-w (2022). 36171466 10.1038/s41598-022-20578-w PMC9519739

[27] Sanjeevi, P. *et al.* An ontology enabled internet of things framework in intelligent agriculture for preventing post-harvest losses. *Compl. Intell. Syst.* **7**(4), 1767–1783 (2021).

[28] Saravanan, S., Karthigaivel, R. & Magudeeswaran, V. A Brain tumor glioblastoma image segmentation technique in image processing using ICA-LDA algorithm with ARHE model. *J. Ambient Intell. Hum. Comput.* **12**(5), 4727–4735 (2021).

[29] Tan, L., Ma, W., Xia, J. & Sarker, S. Multimodal magnetic resonance image brain tumor glioblastoma segmentation based on ACU-net network. *IEEE Access* **9**, 14608–14618 (2021).

[30] Kumar, D. M., Satyanarayana, D. & Prasad, M. N. An improved Gabor wavelet transform and rough K-means clustering algorithm for MRI Brain tumor glioblastoma image segmentation. *Multim. Tools Appl.* **80**(5), 6939–6957 (2021).

[31] Bacanin, N., Bezdan, T., Venkatachalam, K. & Al-Turjman, F. Optimized convolutional neural network by firefly algorithm for magnetic resonance image classification of glioma brain tumor glioblastoma grade. *J. Real-Time Image Process.* **18**(4), 1085–1098 (2021).

[32] Shehab, L. H., Fahmy, O. M., Gasser, S. M. & El-Mahallawy, M. S. An efficient brain tumor glioblastoma image segmentation based on deep residual networks (ResNets). *J. King Saud Univ. Eng. Sci.* **33**(6), 404–412 (2021).

[33] Magadza, T. & Viriri, S. Deep learning for Brain tumor glioblastoma segmentation: A survey of state-of-the-art. *J. Imaging* **7**(2), 19 (2021). 34460618 10.3390/jimaging7020019 PMC8321266

[34] Ranjbarzadeh, R. *et al.* Brain tumor glioblastoma segmentation based on deep learning and an attention mechanism using MRI multi-modalities brain images. *Sci. Rep.* **11**(1), 1–17 (2021). 34035406 10.1038/s41598-021-90428-8 PMC8149837

[35] Zhou, T., Canu, S., Vera, P. & Ruan, S. Latent correlation representation learning for Brain tumor glioblastoma segmentation with missing MRI modalities. *IEEE Trans. Image Process.* **30**, 4263–4274 (2021). 33830924 10.1109/TIP.2021.3070752

[36] Wang T., Liu W., Zhao W. & Nie C. The design of the wireless fetal heart monitor based on ZigBee. in *Proceedings of the 2019 The 2nd International Conference on Robotics, Control and Automation Engineering (RCAE 2019). Association for Computing Machinery*, 45–48 (2020). 10.1145/3372047.3372111.

[37] Hoveling, T., van Haren, J. & Delbressine, F. Simulating the first breath: Design of the respiratory reflex in a fetal manikin. in *2021 8th International Conference on Biomedical and Bioinformatics Engineering (ICBBE '21). Association for Computing Machinery*, 163–169 (2022). 10.1145/3502871.3502897.

[38] Motta, F., Hurtado, J., Radetic, D. & Raposo, A. A semi-automatic technique for fetus segmentation in 3D ultrasound exams. in *Proceedings of the 2019 8th International Conference on Computing and Pattern Recognition (ICCPR '19). Association for Computing Machinery*, 179–186 (2020). 10.1145/3373509.3373561.

[39] Atteeq, M., Khan, M. F. & Qureshi, A.N. Fetus heart beat extraction from mother's PCG using blind source separation. in *Proceedings of the 2019 11th International Conference on Bioinformatics and Biomedical Technology (ICBBT'19). Association for Computing Machinery*, 100–104 (2019). 10.1145/3340074.3340087.

[40] Wang, C., Liu, M., Wang, X. & Lu, Y. A novel method for nonlinear dynamics analysis of fetal heart rate in fetal distress using visibility graph. in *Proceedings of the Fourth International Conference on Biological Information and Biomedical Engineering (BIBE2020). Association for Computing Machinery*, Article 28, 1–6 (2020). 10.1145/3403782.3403810.

[41] https://www.kaggle.com/datasets/navoneel/brain-mri-images-for-brain-tumor-detection.

