# Author Correction: Exploring fetal brain tumor glioblastoma symptom verification with self organizing maps and vulnerability data analysis

**DOI:** 10.1038/s41598-024-60571-z

**Published:** 2024-04-26

**Authors:** Suresh Kumar Natarajan, Jayanthi S, Sandeep Kumar Mathivanan, Hariharan Rajadurai, Benjula Anbu Malar M.B, Mohd Asif Shah

**Affiliations:** 1 School of Computer Science and Engineering, JAIN (Deemed-to-be University), Ramanagara, India; 2 Department of Information Technology, Guru Nanak Institute of Technology, Ibrahimpatnam, Hyderabad, Telangana India; 3 School of Computer Science and Engineering, Galgotias University, Greater Noida, 203201 Uttar Pradesh India; 4 School of Computing Science and Engineering, VIT Bhopal University, Bhopal–Indore Highway Kothrikalan, Sehore, MP India; 5 School of Computer Science Engineering and Information Systems, Vellore Institute of Technology, Vellore, Tamil Nadu India; 6 Kebri Dehar University, Kebri Dehar, 250, Somali Ethiopia; 7 Centre of Research Impact and Outcome, Chitkara University Institute of Engineering and Technology, Chitkara University, Rajpura, 140401 Punjab India; 8 Division of Research and Development, Lovely Professional University, Phagwara, 144001 Punjab India

Correction to: *Scientific Reports* 10.1038/s41598-024-59111-6, published online 16 April 2024

The original version of this Article contained an error in the spelling of the author Benjula Anbu Malar M.B which was incorrectly given as Benjula Anbu M.B.

The original Article has been corrected.

